# Junb controls lymphatic vascular development in zebrafish via miR-182

**DOI:** 10.1038/srep15007

**Published:** 2015-10-13

**Authors:** Kristin Kiesow, Katrin Bennewitz, Laura Gutierrez Miranda, Sandra J. Stoll, Bettina Hartenstein, Peter Angel, Jens Kroll, Marina Schorpp-Kistner

**Affiliations:** 1 Division of Signal Transduction and Growth Control, DKFZ-ZMBH Alliance, German Cancer Research Center (DKFZ), Heidelberg, D-69120, Germany; 2 Department of Vascular Biology and Tumor Angiogenesis, Center for Biomedicine and Medical Technology Mannheim (CBTM), Medical Faculty Mannheim, Heidelberg University, Mannheim, D-68167, Germany; 3 Division of Vascular Oncology and Metastasis, German Cancer Research Center (DKFZ), Heidelberg, D-69120, Germany

## Abstract

JUNB, a subunit of the AP-1 transcription factor complex, mediates gene regulation in response to a plethora of extracellular stimuli. Previously, JUNB was shown to act as a critical positive regulator of blood vessel development and homeostasis as well as a negative regulator of proliferation, inflammation and tumour growth. Here, we demonstrate that the oncogenic miR-182 is a novel JUNB target. Loss-of-function studies by morpholino-mediated knockdown and the CRISPR/Cas9 technology identify a novel function for both JUNB and its target miR-182 in lymphatic vascular development in zebrafish. Furthermore, we show that miR-182 attenuates *foxo1* expression indicating that strictly balanced Foxo1 levels are required for proper lymphatic vascular development in zebrafish. In conclusion, our findings uncover with the Junb/miR-182/Foxo1 regulatory axis a novel key player in governing lymphatic vascular morphogenesis in zebrafish.

The blood and lymphatic vasculature is hallmarked by a high degree of conservation across vertebrates such as human, mouse as well as zebrafish with regard to structure and function^1^. A necessary prerequisite for proper vessel development is a strictly balanced control of gene expression^2^. We have previously reported via loss-of-function approaches in mice that the AP-1 member JUNB is a critical transcriptional regulator of vascular processes. Complete ablation of *Junb* provokes early embryonic lethality due to (i) impaired neoangiogenesis of the decidua, (ii) impaired angiogenesis of the placental labyrinth and (iii) a remodelling failure of the primary vascular plexus of the yolk sac^3^. Moreover, endothelial cell-specific *Junb* abrogation results in a similar phenotype with retarded embryos that die around E10 and display a disorganized vasculature with aberrant branching and dilated vessels^4^. Subsequent work revealed that the function of JUNB in these processes is mediated by transcriptional activation of its targets *Vegfa*^5^, *Cbf*β and *Mmp13*^4^ that act as important regulators of angiogenesis upon cellular hypoxia. The so far identified JUNB target genes implicated in vascular processes and homeostasis^6^ are without exception all positively regulated by JUNB. Nevertheless, JUNB is known as a context-dependent transcriptional regulator with positive and negative regulatory functions^7^,^8^. However, it is not known whether negative regulatory functions of JUNB are equally required for vascular development. Recently, AP-1 members were found to regulate microRNAs (miRNAs), including miR-21^9^, miR-101^10^, miR-144^11^, miR-155^12^ and miR-203^13^ that initiate mRNA decay or inhibit protein translation and, thus, function as fine-tuners of gene expression. Albeit miR-21 and miR-155 can act as modulators of vessel remodelling and vascular disease^14^,^15^,^16^ these microRNAs are not considered as so-called angiomiRs^17^, an emerging group of micro-RNA that is critically involved in angiogenic signalling pathways *in vivo*. MiR-126 was the first angiomiR shown to be required for angiogenesis and vessel integrity in both mice and zebrafish^18^,^19^. In addition, morpholino-mediated knockdown approaches in zebrafish have underscored the importance of micro-RNAs for vascular development^20^,^21^.

Until now, a functional link of specifically JUNB-regulated microRNAs and vascular development is still lacking. In this study, we identify JUNB and a novel JUNB-dependent microRNA, miR-182, as essential regulators for proper lymphatic vascular development in zebrafish.

## Results

### Expression of miR-182 is JUNB-dependently regulated

**Figure 1:**
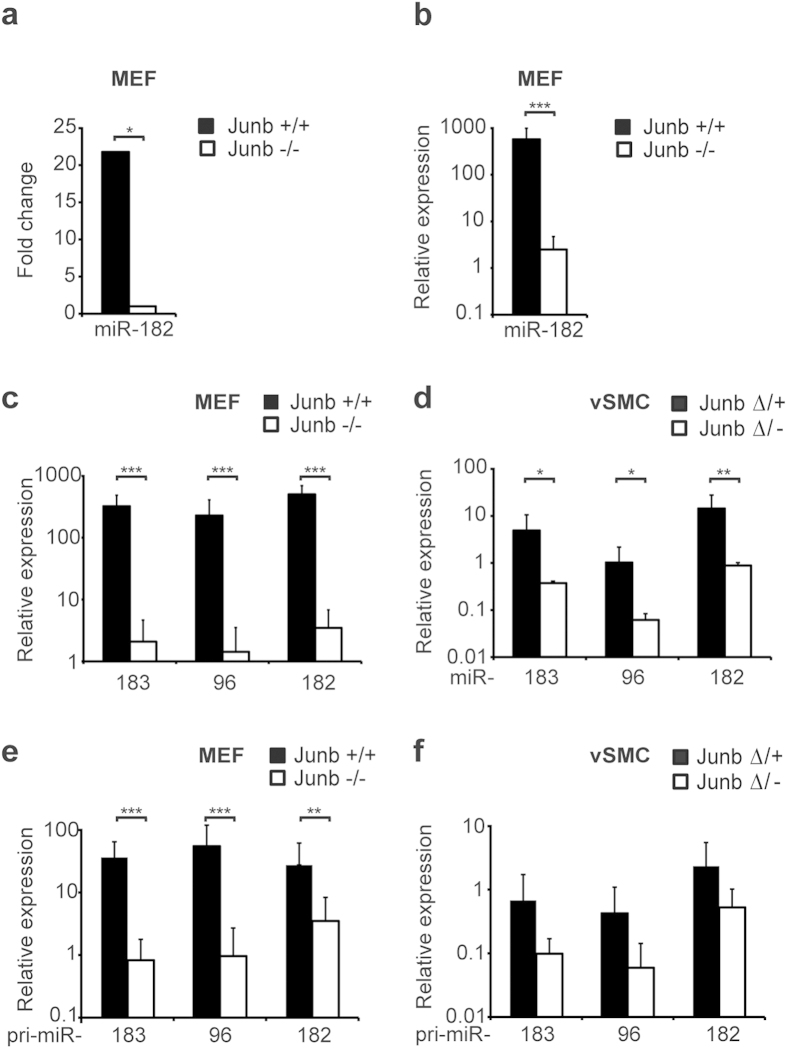
miR-182 is JUNB-dependently expressed. (a) Differential *miR*-*182* expression was identified by miRNA expression profiling of two independent wild type and *Junb*-deficient MEF clone pairs. *limma adjp < 0.05. For details, please refer to Supplementary Methods. (**b**) Validation of mature *miR-182* expression in wild type (+/+) and *Junb*-deficient (−/−) MEFs (n = 5) as determined by Taqman miRNA assay. (**c,d**) Taqman miRNA assays were performed for mature *miR-183, miR-96, miR-182* in wild type and *Junb* knockout (**c**) MEFs (n = 5) and (**d**) vascular smooth muscle cells (vSMCs, n = 3–5). Expression of mature miRNAs was normalized to *U6 snRNA* and plotted relative to *miR-182* expression in *Junb*-deficient cells. (**e,f**) Expression of primary *miR-183,* -*96,* -*182* transcripts was determined by pri-miR-specific Taqman-based qRT-PCR in *Junb* knockout versus wild type (**e**) MEFs (n = 4–8) and (**f**) vSMCs (n = 3–6). Expression levels were normalized to *Hprt* and *B2m* and plotted relative to *pri-miR-182* in *Junb*-knockout cells. (**a–f**) Mean ± SD. ***P < 0.001, **P < 0.01, *P < 0.05, Unpaired Student’s t-test.

In order to identify JUNB-dependently regulated microRNAs, we subjected wild type (*wt*) and *Junb*-deficient (*Junb*^−/−^) mouse embryonic fibroblasts (MEFs)^22^ to a global miRNA expression profiling and identified miR-182. *MiR-182* expression was validated in three distinct *Junb*^−/−^ and *wt* MEF clone pairs by a Taqman miRNA assay, proving that *miR-182* is consistently lost or strongly reduced upon *Junb* ablation (Fig. 1a,b). Recent publications have shown that *miR-182* is a member of the miRNA cluster *miR-183~96~182* likely co-transcribed as a single precursor^23^. Analysis of the expression pattern of the entire cluster in MEFs revealed that the expression of all three mature miRNAs strictly depends on JUNB (Fig. 1c). Furthermore, expression analyses in primary vascular smooth muscle cells (vSMCs) and primary endothelial cells (ECs) derived from control or conditional *Junb* knockout mice (*Junb*^Δ/−^ *Col1*^α*2*-^cre)^24^ confirmed that JUNB is essential for the full expression of the *miR-183~96~182* cluster in vSMCs (Fig. 1d) and in ECs, albeit expression levels in ECs are rather low (data not shown). In order to exclude that the impaired expression of the whole miRNA cluster is due to aberrant miRNA processing in the absence of JUNB, we determined the expression levels of the primary transcripts. Pri-miR-specific Taqman assays showed that the primary transcripts were similarly dependent on JUNB when compared to the mature miRNAs (Fig. 1e,f) indicating that loss of *Junb* rather affects transcription than post-transcriptional processing of the miRNA cluster.

### Silencing of *junb* in zebrafish causes failure in parachordal lymphangioblast formation

**Figure 2:**
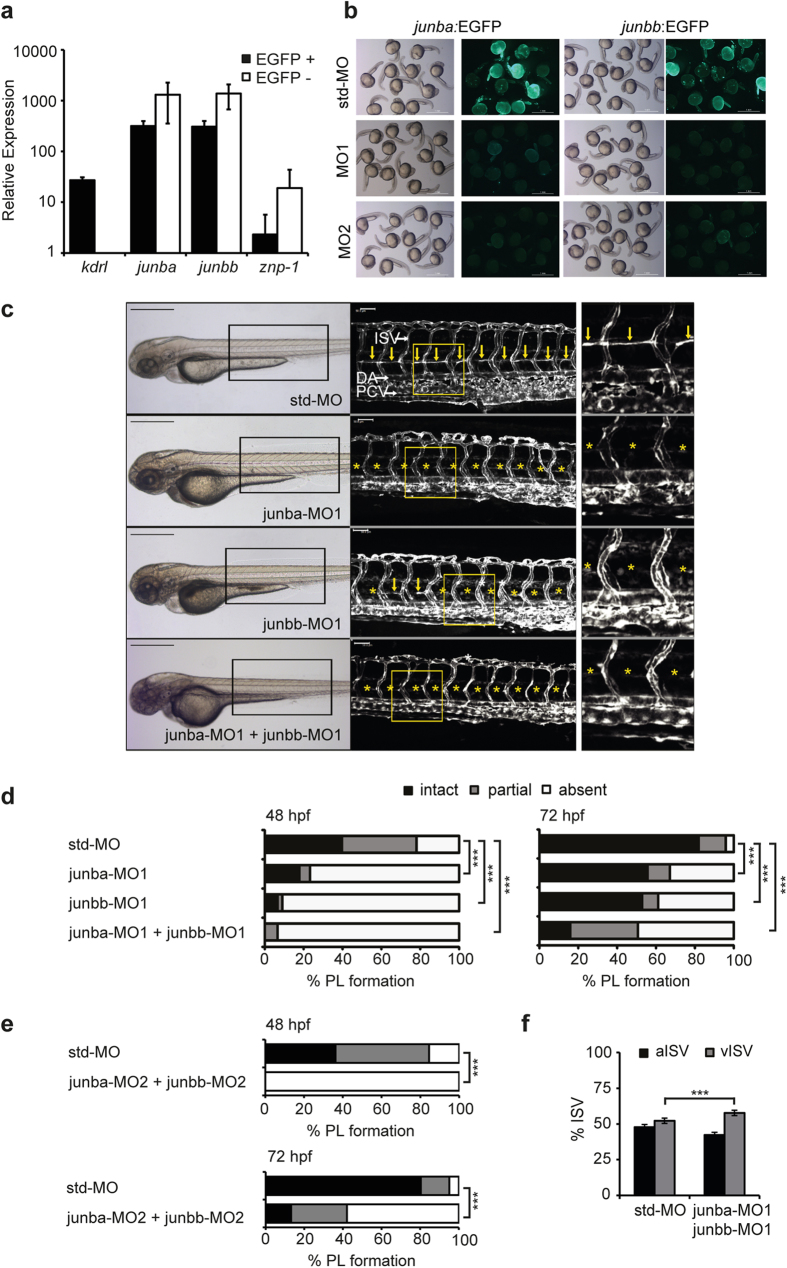
Junb knockdown in zebrafish causes failure in PL formation. (**a**) Expression analysis of cDNA isolated from EGFP + FAC-sorted cells from 24 hpf *Tg*(*fli1:EGFP*)^*y1*^ embryos. *Junba* and *junbb* are expressed in ECs at levels similar to the EC marker *kdrl*. The strong enrichment of *kdrl* in the GFP + and of the neuronal marker *znp-1* in the GFP- fraction, respectively, proves a proper FAC-sorting. Error bars indicate Mean +/- SD (**b**) Wild type zebrafish embryos co-injected with 100 pg of a linearized *junba*:EGFP or *junbb*:EGFP reporter plasmid and 2 ng of the respective experimental or standard morpholino (std-MO) as indicated were assayed for reporter EGFP expression at 24 hpf. Embryos injected with either *junb*:EGFP-reporter construct and std-MO display strong EGFP expression, while either *junba* or *junbb* morpholino completely blocked the EGFP signal, respectively. Panel displays representative embryos from each injection group. (**c**) Left panel, bright field images of representative embryos at 72 hpf injected with the indicated morpholino: std-MO (4 ng, n = 104), *junba*-MO1 (2 ng, n = 115), *junbb*-MO1 (2 ng, n = 92), *junba*-MO1 + *junbb*-MO1 (2 ng + 2 ng, n = 108). Middle panel, confocal images of trunk vasculature in *Tg*(*fli1:EGFP*)^*y1*^ embryonic region marked by rectangle on the left. Right panel, enlarged view of trunk region marked by the yellow rectangle in the middle panel. DA, dorsal aorta; PCV, posterior cardinal vein; ISVs, intersegmental vessels; PL is marked with yellow arrows or if absent with asterisks. Panels are lateral view; dorsal is up, anterior to the left. Scale bars represent 500 μm (black) and 50 μm (white). (**d,e**) Percentage of PL defects in embryos shown in (**c**) at 48 and 72 hpf and for a non-overlapping second set of *junb*-specific translation-blocking MOs (MO-2). std-MO (n = 58), *junba*-MO2 + *junbb*-MO2 (n = 76). Sum of 16 hemisegments per embryo was categorized into groups with no, partial or complete absence of PL. ***P < 0.001, Mann-Whitney U-test. (**e**) Quantification of arterial and venous ISVs (aISV, vISV) given as % aISV and vISV in *Tg*(*fli1:EGFP*)^*y1*^ zebrafish embryos injected with indicated morpholinos at 72 hpf. Mean ± SD, n = 50-60 embryo/group. ***P < 0.001, Student’s t-test.

Since JUNB is a critical regulator for proper angiogenesis in mice^3^,^4^,^5^, we wondered whether the newly identified Junb*-*dependent *miR-182* is required for vascular development as well. To address this question, we chose the morpholino-mediated knockdown of either *junb* or *miR-182* in zebrafish embryos. The mammalian *Junb* gene has two orthologs in zebrafish–*junba* and *junbb*^25^ coding for proteins with a sequence identity of 75.6%–and both transcripts are expressed at similar levels with increasing abundance from 24 to 96 hours post fertilization (hpf; see Supplementary Fig. S1). Monitoring the expression pattern of both *junb* transcripts by whole-mount *in situ* hybridization of 30 hpf embryos confirmed previously published data^26^,^27^, for example high *junba* and *junbb* expression in the eye (see Supplementary Fig. S1). In addition, *junba* and *junbb* transcripts were seen in the proctodeum/cloaca. Since a weak expression of *junbb* was observed in the mesoderm and vasculature (see Supplementary Fig. S1) we analysed *junb* expression in ECs isolated by flow cytometer sorting of EGFP-positive cells from 24 hpf *Tg*(*fli1:EGFP*)^*y1*^ zebrafish embryos (Fig. 2a). qRT-PCR analysis revealed relatively high expression levels of *junb* in the EGFP-negative cell fraction most likely due to the prominent expression of *junb* in the retina and brain (see Supplementary Fig. S1). Yet, *junba* as well as *junbb* transcripts were abundantly expressed in ECs and the relative expression level was even higher than the level of the EC-specific marker *kdrl* (Fig. 2a). Thus, we chose the *Tg*(*fli1:EGFP*)^*y1*^ zebrafish reporter line for the knockdown approaches and, due to the lack of introns in both *junb* genes, we injected ATG-blocking morpholinos (MOs) against each individual *junba*, *junbb* and both *junb* mRNAs (see Supplementary Fig. S1).

In the absence of validated commercially available antibodies that are able to detect and discern zebrafish Junba and Junbb proteins, we opted for an alternative approach to determine the degree of protein knockdown for each paralog. We created in-frame EGFP reporter constructs containing the Junb paralog-specific morpholino binding sites (see Supplementary Fig. S1), and co-injected each MO with the corresponding linearized DNA construct in single cell embryos. When 100 pg of *junba*-EGFP or *junbb*-EGFP were injected most embryos displayed strong EGFP expression at 24 hpf (data not shown). Co-injection with 2 ng of the experimental morpholinos *junba*-MO1/2 with *Junba*-EGFP or of ^junbb^-MO1/2 with *Junbb*-EGFP completely suppressed EGFP expression, while std-MO had little effect on reporter expression (Fig. 2b), indicating that the ^Junb^-specific MOs evoke an efficient and specific translation inhibition.

Based on the data we used 2 ng of each MO for further experiments. *Junba* and *junbb* morphants did not show any gross morphological aberrations. Formation of the major vessels in the zebrafish trunk such as the dorsal aorta (DA), posterior cardinal vein (PCV), intersegmental vessels (ISV), parachordal lymphangioblasts (PL) and dorsal longitudinal anastomotic vein (DLAV) was examined by confocal microscopy and found to be normal for embryos injected with a standard control morpholino (std-MO, Fig. 2c). By contrast, morphants injected either with *junba*-MO1, *junbb*-MO1 or the combination of both exhibited an almost complete lack of parachordal lymphangioblasts (PL, Fig. 2c). Other vessels in the zebrafish trunk such as the DA, PCV, ISV and DLAV did develop normally and were mostly unaltered except for some slight individual variations. At 48 hpf, the PL was entirely absent in 77%, 91%, and 94% of morphants injected with *junba*-MO1, *junbb*-MO1, or both, respectively, versus 22% of control embryos (Fig. 2d, left panel). By 72  hpf, formation of the PL should be completed^26^. At that time, 82% of investigated control embryos showed a complete PL, 55% and 53% of *junba* and *junbb* single morphants, respectively, but only 16% of *junb* double morphants exhibited a complete PL (Fig. 2d, right panel). Data obtained with a second non-overlapping set of *junb*-specific MOs (MO2; see Supplementary Fig. S1) gave identical results (Fig. 2e). By 72 hpf, again only 13% of *junb* double morphants injected with *junba-*MO2 and *junbb*-MO2 developed a complete PL (Fig. 2e, lower panel).

In order to exclude that the defects in PL formation are secondary to a disrupted blood circulation eventually due to a heart failure, we monitored the beating heart of 48 hpf embryos (see Supplementary Videos 1 to 4), the blood flow and the distribution of arterial and venous intersegmental vessels in the trunk region of 72 hpf embryos (see Supplementary Videos 5 to 8). Video analysis revealed that in *junb* double morphants similar to control morphants, the heart appeared normal and blood cells constantly travelled through the vascular network in the trunk (see Supplementary Videos 1 to 8). Quantitative analysis of the arterial-venous identity of intersegmental vessels elicited a ratio of aISVs to vISVS of 48:52% for morphants injected with a standard morpholino (Fig. 2f). In *junb* double morphants this ratio was slightly shifted towards 43:57% in favour for the veins (Fig. 2f). In summary, although *junb* double morphants display a slight increase in the number of venous ISVs, they are devoid of any obvious defects in blood circulation, which might interfere with PL formation.

### Knockout of *junb* in zebrafish phenocopies lymphatic vascular phenotype of *junb* morphants

**Figure 3:**
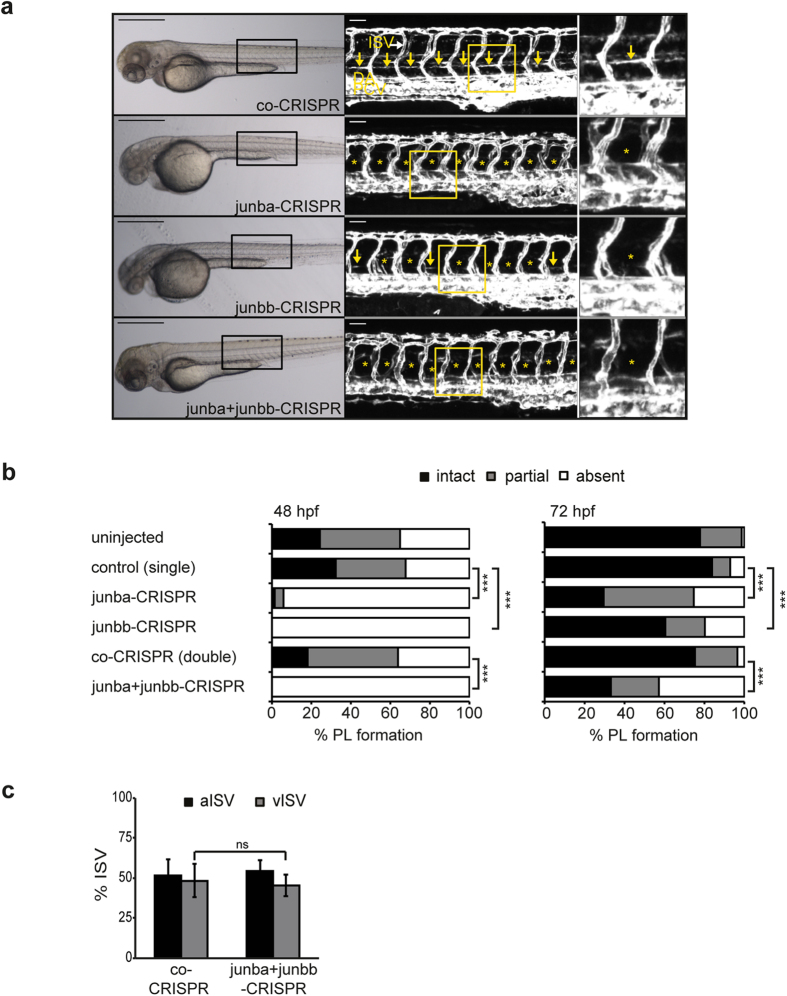
Junb knockout in zebrafish phenocopies the MO-mediated Junb knockdown PL phenotype. (**a**) Left panel: Bright field images of representative embryos at 72 hpf injected with the indicated CRISPR gRNA: co-CRISPR (400 pg), *junba*-CRISPR (250 pg), *junbb*-CRISPR (250 pg), *junba*-CRISPR + *junbb*-CRISPR (200 pg + 200 pg). Middle panel: Confocal images of trunk vasculature in *Tg*(*fli1:EGFP*)^*y1*^ embryonic region marked by rectangle on the left. Right panel, enlarged view of trunk region marked by the yellow rectangle in the middle panel. DA, dorsal aorta; PCV, posterior cardinal vein; ISVs, intersegmental vessels; PL marked with yellow arrows or if absent with asterisks. Panels are lateral view; dorsal is up, anterior to the left. Scale bars represent 500 μm (black) and 50 μm (white). (**b**) Percentage of PL defects in embryos (at 48 and 72 hpf) injected with CRISPR gRNAs as described in (**a**) against either *junba* (n = 68), *junbb* (n = 94) or in combination against both (n = 150) versus embryos injected with control CRISPR at corresponding doses of 250 pg (n = 99) or 400 pg (co-CRISPR, n = 105). Sum of 16 hemisegments per embryo was categorized into groups with no, partial or complete absence of PLs. ***P < 0.001, Mann-Whitney U-test. (**c**) Quantification of arterial and venous ISVs (aISV, vISV) given as % aISV and vISV in *Tg*(*fli1:EGFP*)^*y1*^ zebrafish embryos at 72 hpf injected with indicated CRISPR gRNAs. n = 40 embryo/group, mean ± SD. **P < 0.01, ns, not significant, Student’s t-test.

To further verify whether loss of Junb causes indeed a failure in lymphatic vessel development in zebrafish, we used a reverse genetics approach applying the CRISPR/Cas9 technology. We generated CRISPR (Clustered Regularly Interspaced Short Palindromic Repeats) guide RNAs (gRNAs) to individually target zebrafish *junba* and *junbb* genomic DNA (see Supplementary Fig. S2). T7 endonuclease I assay and High Resolution Melting (HRM) analyses proved that the *junb* CRISPR gRNAs were highly efficient at specifically mutating the two *junb* loci since six to eight out of eight randomly selected embryos carried mutations (see Supplementary Fig. S2). Injecting CRISPR gRNAs together with *Cas9* mRNA did neither affect overall embryonic morphology, nor blood vessel formation, but provoked a specific failure in lymphatic vessel development as observed in *junb* morphants (Fig. 3). At 48 hpf, the PL was entirely absent in 94% and 100% of *junba*- and *junbb*-CRISPR gRNA-injected embryos compared to 35% of control-CRISPR gRNA-injected or untreated embryos (Fig. 3b, left panel). By 72 hpf, 78% of uninjected and 84% of control CRISPR gRNA-injected embryos formed a complete PL. In contrast, only 30% and 60% of *junba*- and *junbb*-CRISPR gRNA-injected embryos, respectively, displayed an intact PL (Fig. 3b, right panel). To test, if combined knockout of both Junb paralogs further enhanced the phenotype we multiplexed the CRISPR guide RNAs for *junba* and *junbb* and found that 67% of double *junba*- and *junbb*-CRISPR gRNA-injected embryos failed to develop an intact PL (Fig. 3b, right panel) by 72 hpf. Quantification of the aISVs versus vISVs revealed a ratio of arteries to veins of 52:48% for embryos injected with a control CRISPR (Fig. 3C). The aISV:vISV ratio in *junba*- and *junbb*-CRISPR gRNA-injected embryos was 55:45% (Fig. 3c). Yet, comparison of percentage vISVs of control CRISPR-injected embryos versus vISVs of *junba*- and *junbb*-CRISPR gRNA-injected embryos revealed no significant difference. In summary, we observed a high correlation between phenotypes of the CRISPR gRNA-injected embryos and *junb* morphants with regard to lymphatic vessel development.

### Knockdown of *miR-182* mimics the lymphatic vascular phenotype of *junb* morphants

**Figure 4:**
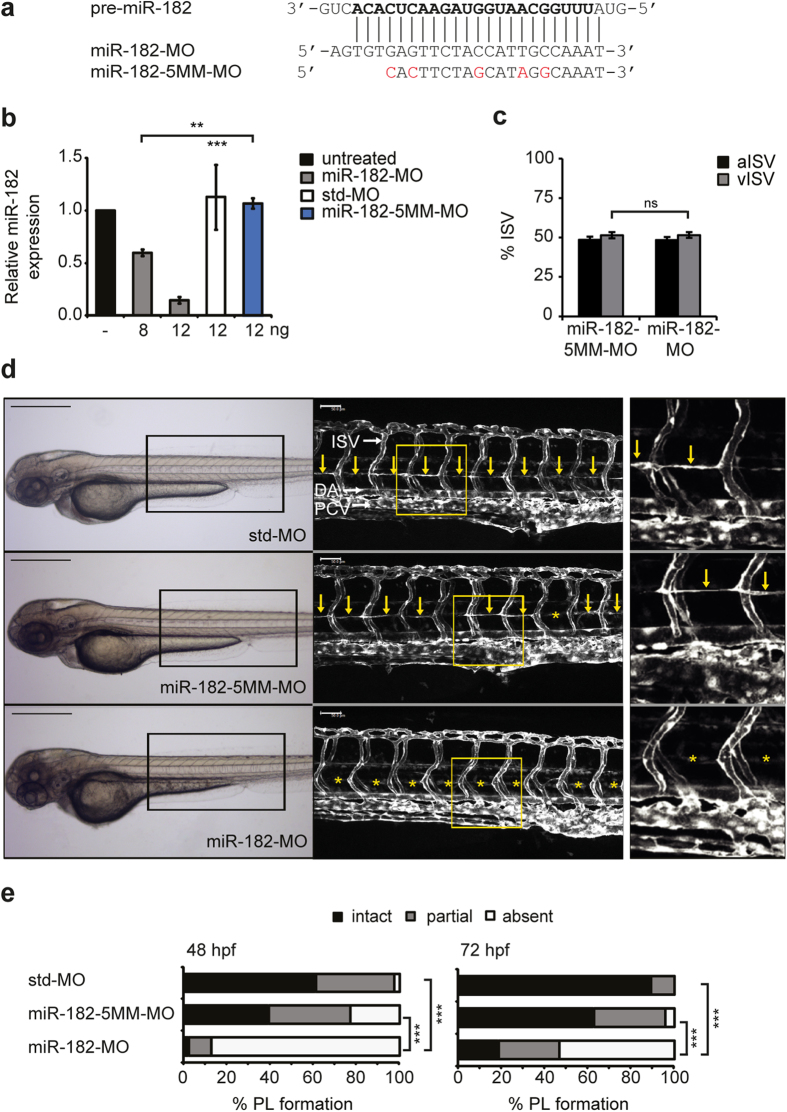
miR-182 expression silencing recapitulates the Junb knockdown PL phenotype. (**a**) Sequences of the guide strand of targeted *pre-miR-182* and of *miR-182* (*miR-182*-MO) and 5-mismatch control (*miR-182*-5MM-MO) morpholinos used. Nucleotides of mature *miR-182* and substituted nucleotides of *miR-182*-5MM-MO are marked in bold and red, respectively. (**b**) Dose-dependent knockdown of *miR-182* as determined by Taqman miRNA assay in 48 hpf zebrafish embryos following injection of *miR-182*-MO, std-MO or *miR-182*-5MM-MO using the indicated MO concentrations. Data are normalized to *U6 snRNA* and expressed as mean ± SEM (n = 3). ***P < 0.001, **P < 0.01, Unpaired Student’s t-test for comparison to *miR-182*-5MM-MO. (**c**) Quantification of arterial and venous ISVs (aISV, vISV) given as % aISV and vISV in *Tg*(*fli1:EGFP*)^*y1*^ zebrafish embryos injected with indicated morpholinos at 72 hpf given as % aISV and % vISV. Mean ± SD. ns, not significant, Student’s t-test. (**d**) Left panel, representative bright field images of zebrafish embryos 72 hpf injected with *miR-182*-MO (12 ng, n = 151) compared to std-MO (12 ng, n = 107) and *miR-182*-5MM-MO (12 ng, n = 139). Middle panel, confocal images of trunk vasculature in respective embryonic region marked by rectangle on the left. Right panel, enlarged view of trunk region marked by the yellow rectangle in the middle panel. DA, dorsal aorta, PCV, posterior cardinal vein and ISV, intersegmental vessels. PL is marked with yellow arrows or if absent with asterisks. Panels are lateral view; dorsal is up, anterior to the left. Scale bars: 500 μm (black) and 50 μm (white). (**e**) Percentage of PL defects in embryos at 48 hpf and 72 hpf. ***P < 0.001, Mann-Whitney U-test.

To investigate whether *miR-182* indeed acts functionally downstream of Junb in zebrafish, we silenced *miR-182* in *Tg*(*fli1:EGFP*)^*y1*^ transgenic zebrafish and used in addition to the standard morpholino a *miR-182*-MO carrying 5-mismatches (*miR-182*-5MM-MO; Fig. 4a) as specificity control. Injection of 12 ng of *miR-182*-MO provoked an 80% reduction of the *miR-182* levels compared to morphants injected with std-MO (Fig. 4b) but did not give rise to unspecific side effects as judged by overall zebrafish morphology (Fig. 4c–e). Confocal microscopy revealed a normal formation of the DA, PCV and ISVs (Fig. 4d) as well as a normal ratio of aISVs:vISVs with 48:52% (Fig. 4c). Remarkably, silencing of *miR-182* mimicked the double knockdown of *junb* with regard to the PL phenotype in both temporal occurrence and strength of the defects (Fig. 4d,e). At 48 hpf, 88% of embryos exhibited a complete lack of the PL upon injection of *miR-182*-MO vs. 3% of the controls. (Fig. 4e, left panel) As the PL was absent in only 23% of *miR-182*-5MM-MO injected embryos and as this defect in *miR-182*-5MM morphants was compensated during development, an off-target effect can be excluded. At 72 hpf, a complete and partial PL was present in 90% of *miR-182*-5MM morphants compared to 100% in control morphants injected with an unrelated std-MO (Fig. 4e, right panel). By contrast, only 46% of *miR-182* morphants were able to develop at least a partial PL. Taken together, knockdown of *miR-182* yields a similar lymphatic vascular phenotype as the loss of *junb* in zebrafish with a specific and striking effect on the formation of the PL.

### Ectopic *miR-182* restores the PL failure evoked upon junb expression silencing

**Figure 5:**
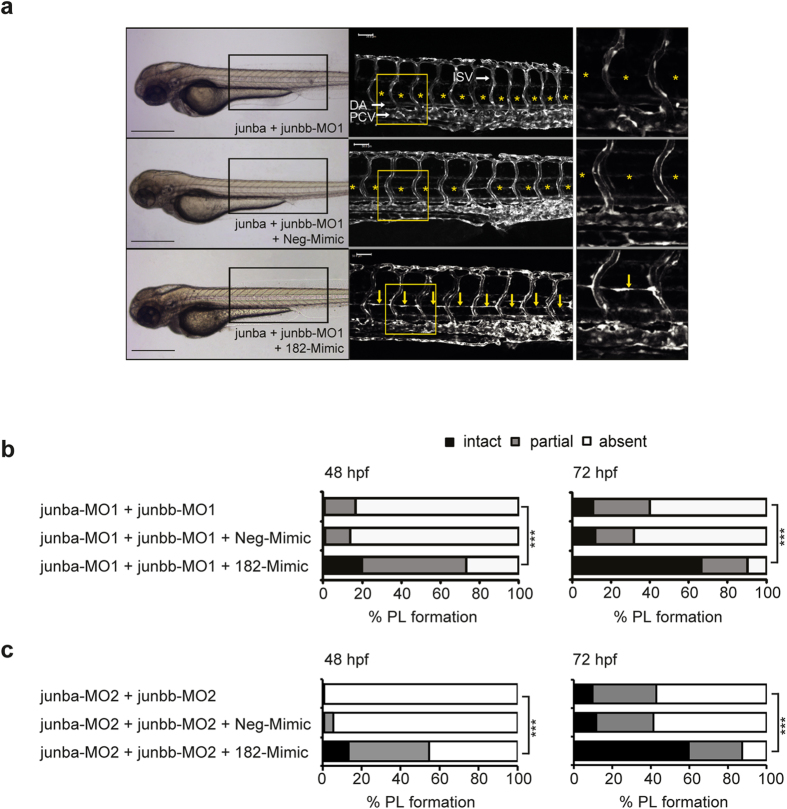
Ectopic miR-182 restores PL formation in Junb morphants. (**a**) Left panel, bright field images showing overall morphology of *junba* and *junbb* (2 + 2 ng) double morphants injected with or without Neg-Mimic (40 pg, n = 127) or 182-Mimic (40 pg, n = 109). Middle panel: Confocal images of indicated trunk region in *Tg*(*fli1:EGFP*)^*y1*^ embryos at 72 hpf. Right panel, enlarged view of trunk region marked by the yellow rectangle in the middle panel. DA, dorsal aorta, PCV, posterior cardinal vein and ISV, intersegmental vessels. PL is marked with yellow arrows or if absent with asterisks. Panels are lateral view; dorsal is up, anterior to the left. Scale bars: 500 μm (black); 50 μm (white). (**b**) Percentage of embryos injected with MOs and Mimics as indicated and (**c**) for a second set of *junb*-MOs: *junba*-MO2 + *junbb*-MO2 (2  ng + 2 ng, n = 112), with or without Neg-Mimic (40 pg, n = 145) or 182-Mimic (40 pg, n = 119). ***P < 0.001, Mann-Whitney U-test.

Next, we examined whether the phenotypes caused by *junb* silencing can be rescued by overexpression of *miR-182*. For this purpose, we co-injected a double-stranded *miR-182* (182-Mimic) or a negative control oligonucleotide (Neg-Mimic) into *junba* + *junbb* double morphants generated by two independent sets of *junba*- and *junbb*-specific MOs (MO1 and MO2). The optimal mimic concentration was established and *miR-182* overexpression was validated by Taqman miRNA assay (see Supplementary Fig. S3). Sole injection of the Neg-Mimic or 182-Mimic itself provoked no apparent phenotype (see Supplementary Fig. S3). Similarly, co-injection of Neg-Mimic did not significantly alter the penetrance of the PL phenotype in *junba/junbb* double morphants (Fig. 5a,b). Most importantly, co-application of the 182-Mimic into the *junba/junbb* double morphants substantially restored the PL formation from 11% to 75% (MO1) or from 0% to 53% (MO2) of embryos with complete and partial PL as quantified at 48 hpf (Fig. 5b, left panel). At 72 hpf, ectopic *miR-182* administration restored the PL in *junba/junbb* double morphants such that now 67% (MO1) and 60% (MO2) of embryos displayed an entirely intact PL compared to 11% and 10 % of *junba/junbb*-MO1 and -MO2 knockdown embryos, respectively (Fig. 5b,c). Thus, data obtained with an independent second set of *junb*-specific MOs (MO2) gave nearly identical results. These findings indicate that *miR-182* acts downstream of Junb and strongly suggest that Junb and *miR-182* act in a common signalling pathway crucial for lymphatic vascular morphogenesis in zebrafish.

### Silencing of either *junb* or *miR-182* inhibits thoracic duct formation in zebrafish

**Figure 6:**
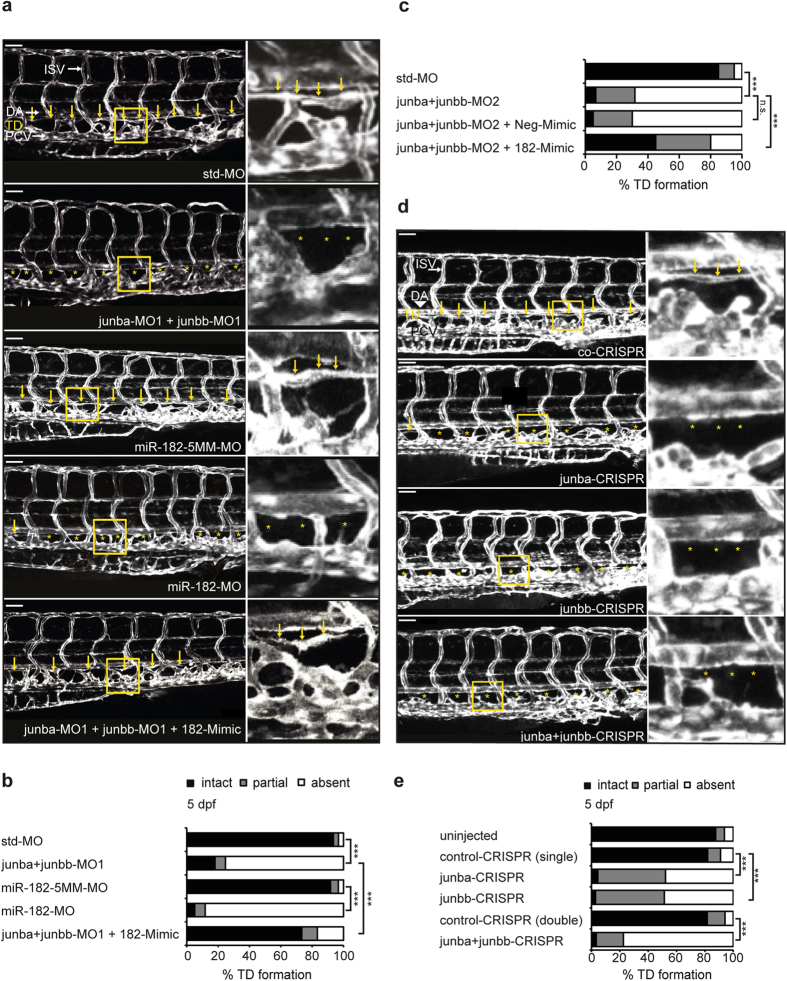
Failure in TD formation of Junb morphants is restored by ectopic miR-182. (**a**) Left panel, confocal images of trunk vasculature of representative larvae at 5 dpf injected with indicated morpholino or mimic: std-MO (4 ng, n = 62), *junba*-MO1 + *junbb*-MO1 (2 ng + 2 ng, n = 60), *miR-182*-5MM-MO (12 ng, n = 59), *miR-182*-MO (12 ng, n = 60), *junba*-MO1 + *junbb*-MO1 + 182-Mimic (2 ng + 2 ng + 40 pg, n = 32). Middle panel, confocal images of indicated trunk region. Right panel, enlarged view of trunk region marked by yellow rectangle in the middle panel. TD is located between dorsal aorta (DA) and posterior cardinal vein (PCV) and is marked with yellow arrows or if absent with asterisks. Panels are lateral view; dorsal is up, anterior to the left. White scale bar: 50 μm. (**b,c**) Percentage of TD defects in larvae for the first (**b**) and the second set of junb-MOs (**c**). (**d**) Left panel, confocal images of trunk vasculature of representative larvae at 5 dpf CRISPR gRNA-injected larvae with indicated *junb*-CRISPR gRNAs or control CRISPR gRNA: *junba*-CRISPR (250 pg gRNA), *junbb*-CRISPR (250 pg gRNA), *junba*-CRISPR + *junbb*-CRISPR (200 pg + 200 pg gRNA) and Co-CRISPR (400 pg gRNA). Labelling and size bar as described in (**a**). (**e**) Percentage of TD defects in larvae at 5 dpf injected with CRISPR gRNAs either against *junba*, *junbb* or a combination of both versus larvae injected with control CRISPR gRNA. Sum of 10 somite segments per embryo was categorized into groups with presence, partial or complete absence of TD. ***P < 0.001, Mann-Whitney U-test.

Although we analysed PL formation at 72 hpf, a time point at which PL formation should be completed^28^, we also investigated the formation of the thoracic duct (TD) at 5 dpf in order to exclude that the PL phenotype is simply caused by a developmental delay. In control zebrafish larvae, injected with a std-MO or a *miR-182*-5MM-MO, the TD was formed in 93% larvae by 5 dpf as a continuous lymphatic vessel (Fig. 6a,b). By contrast, only 17% of *junba/junbb* double morphants and 11% of zebrafish larvae with silenced *miR-182 expression* developed a continuous TD by 5 dpf, respectively (Fig. 6a,b). These data indicate that development of the lymphatic vascular system was not delayed but completely aborted upon knockdown of *junb* or *miR-182*. In line with our previous rescue approach, ectopic expression of the 182-Mimic in *junba/junbb-MO1* double morphants completely restored TD formation by 5 dpf in 73% of larvae (Fig. 6a,b), which could be re-capitulated with a second set of *junb* MOs (Fig. 6c), supporting the concept that *miR-182* is a Junb target required for lymphatic vascular morphogenesis in zebra-fish. Importantly, *junba*- and *junbb*-CRISPR gRNA-injected larvae also fail to form an intact TD. While around 80% of control CRISPR gRNA-injected larvae developed a continuous TD by 5 dpf, only 4% and 7% of *junba*- and *junbb*-CRISPR gRNA-injected larvae, respectively, displayed an intact TD. Moreover, combined injection of both *junba*- and *junbb*-CRISPR gRNAs further enhanced the phenotype resulting in 97% larvae lacking an intact TD (Fig. 6d).

### Strict miR-182–mediated control of foxo1 expression is required for PL formation in zebra-fish

**Figure 7:**
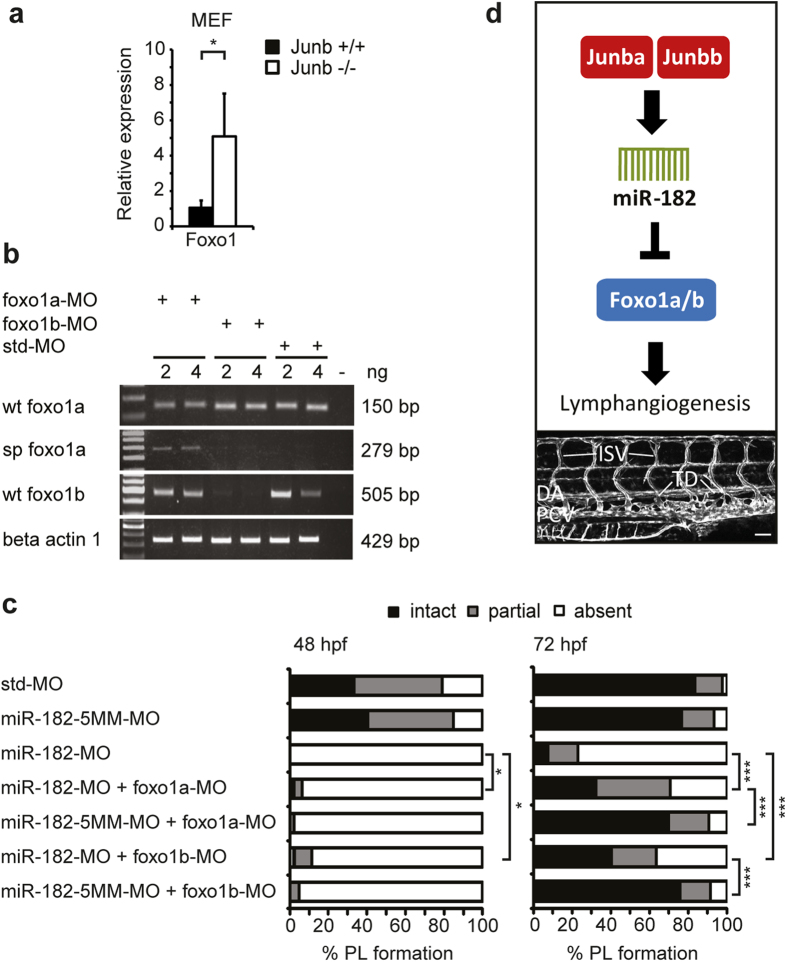
Foxo1 knockdown rescues the PL formation failure of miR-182 morphants. (**a**) Expression analysis by qRT-PCR revealed an inverse correlation of *foxo1* with *miR-182* expression in wild type vs. *Junb*-deficient MEFs. Mean ± SD (n = 3). *P < 0.05, Unpaired Student’s t-test. (**b**) RT-PCR of *foxo1a*-MO, *foxo1b*-MO and std-MO (2 ng and 4 ng) injected zebrafish embryos at 48 hpf detecting the wt *foxo1* transcripts from std-MO-injected embryos, a larger *foxo1a* transcript (*sp foxo1a*) upon injection of the exon skipping *foxo1a*-MO and no *foxo1b* transcript upon injection of its specific MO. For details please refer to Supplementary Fig. S4. Data represent results from one of four independent experiments with similar results. *Actb1* served as a loading control. (**c**) Percentage of PL formation at 48 hpf and 72 hpf in embryos injected with MOs as indicated. n = 82 (12 ng *miR-182*-MO), n = 248 (12 ng *miR-182*-MO + 2 ng *foxo1a*-MO), n = 118 (12 ng *miR-182*-5MM-MO + 2 ng *foxo1a*-MO), n = 163 (12 ng *miR-182*-MO + 2 ng *foxo1b*-MO), n = 103 (12 ng *miR-182*-5MM-MO + 2 ng *foxo1b*-MO). n = 181 (14 ng std-MO), n = 77 (12 ng *miR-182*-5MM-MO). ***P < 0.001, *P < 0.05, Mann-Whitney U-test. (**d**) Scheme for novel regulatory axis in developmental lymphangiogenesis in zebrafish.

Since loss of *miR-182* is impairing proper PL and TD formation (Figs. 4 and 6), we hypothesized that overexpression of a potential *miR-182* target is causing the observed lymphatic vascular malformations. A few known regulators of PL formation, including Cortactin^29^, Elmo1^30^ and Netrin 1a^31^,^32^ could be targeted by *miR-182* according to *in silico* target prediction software. Yet, only silencing of those targets hampers PL formation while their overexpression has no impact^29^,^30^,^31^,^32^. Interestingly, the transcription factor Foxo1 was recently validated as a *miR-182* target in zebrafish implicated in osteoblast proliferation and differentiation^33^. In addition, Foxo1 acts as a negative regulator of angiogenesis by interfering with endothelial cell migration, sprouting and tube formation^34^. In support of the assumption that *foxo1* is one important downstream target of Junb and miR-182, we identified significantly increased *foxo1* mRNA levels in *junb*-deficient MEFs (Fig. 7a). As proof of our hypothesis that *miR-182*-mediated silencing of *foxo1* is required for normal PL formation in zebrafish, we aimed to co-inject *foxo1* morpholinos into *miR-182* morphants to rescue the phenotype of *miR-182* morphants. Zebrafish have two *foxo1* para-logs: *foxo1a* and *foxo1b*. To knockdown *foxo1a* we used a morpholino blocking the intron 2- exon 3 splice site of *foxo1a* generating an exon-skipping splice product referred to as *sp foxo1a* (Fig. 7b and see Supplementary Fig. S4). *Foxo1b* was silenced using a splice-blocking morpholino targeting the exon 1- intron 1 splice site and, thus, abolishing any mature *foxo1b* transcript (Fig. 7b, and see Supplementary Fig. S4). Since zebrafish embryos injected with 4 ng of either morpholino exhibited already minor morphological alterations (data not shown), subsequent experiments were performed with 2 ng of either *foxo1a*- or *foxo1b*-MO. Silencing of either *foxo1a* or *foxo1b* did neither influence *miR-182* expression, nor ISV and DLAV-formation (Supplementary Fig. 4B) but in agreement with previous findings in *Xenopus laevis*^35^ caused a mild developmental retardation with an initial lack of PL formation at 48 hpf (Fig. 7C, Supplementary Fig. 4). Interestingly, PL formation in *foxo1* morphants was regained at 72 hpf and no longer significantly different from control morphants (Fig. 7C, Supplementary Fig. 4). Yet, the combined silencing of *foxo1a* and *foxo1b* resulted in an impairment of PL formation with only 40% of morphants displaying an intact PL at 72 hpf (Supplementary Fig. S4). Thus, we attempted to rescue the PL failure in *miR-182* morphant embryos by co-injection of a submaximal dose of either *foxo1a*- or *foxo1b*-MO. Importantly, co-silencing of either *foxo1* paralog in *miR-182* morphants reduced the penetrance of the PL failure at 72 hpf from formerly 77% to 29% or to 37% when either *foxo1a* or *foxo1b,* respectively, was suppressed (Fig. 7c). PL failure frequency of embryos co-injected with the *miR-182*-5MM-MO and either *foxo1a*- or *foxo1b*-MO was 9% and about similar to std-MO injected embryos. These findings indicate that *foxo1* is one major downstream target of miR-182 and that strictly balanced Foxo1 levels are required for proper lymphatic vascular morphogenesis in zebrafish (Fig. 7d).

## Discussion

This study identifies Junb as a novel lymphangiogenic transcription factor and its newly discovered target *miR-182* as a novel lymphangiomiR as being required to attenuate *foxo1* expression and to allow the development of the lymphatic vasculature in zebrafish. Comparative miRNA expression profiling of *Junb* MEFs revealed that *miR-182* expression depends on Junb presence. *MiR-182* is abundantly expressed in neurosensory cells in the eye, ear and nose^36^ and implicated in retinal development^37^,^38^. MiR-182 plays also a central role in the physiological regulation of IL-2-driven helper T cell-mediated immune responses^39^. For breast and colorectal cancer entities it has been shown that miR-182 promotes proliferation and invasion^40^,^41^. Yet so far, miR-182 has neither been associated with Junb or AP-1 nor with a function in vascular development.

Our functional analyses for Junb and miR-182 in zebrafish revealed a novel essential role for both in lymphatic vascular development. While mammals only have one *junb* gene, encoding a transcriptional regulator that is crucial for the development^3^,^4^ and homeostasis of the blood vasculature^6^, the zebrafish has two gene paralogs, *junba* and *junbb*. In line with a previous report on tissue regeneration in zebrafish showing that Junba and Junbb are both physiologically active proteins^25^, MO-mediated silencing as well as CRISPR/Cas9-mediated mutation of either *junba* or *junbb* indicated an essential function for Junba and Junbb on regulating lymphangiogenesis in zebrafish. Clearly, simultaneous ablation of both paralogs almost completely abrogated PL formation. Hence, we conclude that both Junb paralogs are required for proper early lymphangiogenesis albeit they may functionally compensate for each other to some extent when development advances. In a previous *junb* knockdown approach by Meder *et al.* this PL phenotype was not reported^27^. Instead, they observed a heart failure characterized by pericardial oedema and disrupted blood circulation due to a progressive failure in cardiac contractility by 36 hpf^27^. Upon monitoring heart function by video microscopy (see Supplementary Videos 1–4) we did not observe such a heart failure. This distinct outcome may be explained by the use of a different knockdown strategy and varying amounts of injected MOs. Meder *et al.* have used a translation blocking MO in wild type zebrafishes that is specific for *junba*^27^ but does not match completely with the *junbb* sequence around translational start site. By contrast, we used the *Tg*(*fli1:EGFP*)^*y1*^ reporter line to monitor vessel formation and targeted each *junb* paralog independently by specific translation blocking morpholinos. These MOs bind either within the 5′UTR (*junba*-MO1 and -MO2; *junbb* MO2) or across the translational start site such as the *junbb*-MO1 that covers the same sequence area as the MO previously reported but matches completely with the *junbb* sequence. Since *junba/b* double morphants also do not display any heart defect (see Supplementary Videos 1–4), still exhibit a proper arterial-venous differentiation of the intersegmental vessels and a robust blood circulation in the trunk we can exclude that the lymphatic phenotype is the result of the previously reported cardiac failure in response to *junb* knockdown^27^. Thus, our data identify Junb as a newly emerging lymphatic vascular regulator, a feature that has not been appreciated so far due to the early embryonic lethality caused by *Junb* ablation in mice. Moreover, the here presented data suggest that Junb may be considered as a further vascular regulator in zebrafish besides *flt4*, *ccbe1* and *gata2* for which the morphant phenotype and phenotype of CRISPR gRNA-injected embryos show a high correlation^42^.

Morpholino-mediated knockdown of *miR-182* provoked a failure in the formation of the PL and, subsequently, of the TD. Thus, this phenotype strongly resembles the phenotype of zebrafish morphants with suppressed Junb and can be considered as phenocopy. Most importantly, ectopic expression of a 182-Mimic in *junb* zebrafish morphant embryos rescued the failure in PL and TD formation to a large extent. The penetrance of the PL phenotype provoked upon *miR-182* silencing was nearly identical to that of combined *junb* silencing, providing strong evidence that *miR-182* is an important functional Junb target.

Recently, Nicenboim *et al.* showed that in zebrafish lymphangiogenesis is initiated from posterior cardinal vein angioblasts (vPCVs), which originate in the lateral plate mesoderm^43^. Endoderm-derived Wnt5b induces LEC specification of these vPCVs and at 23 hpf these cells mostly give rise to either parachordal cells (PACs) or venous intersegmental vessels^43^. Post sprouting, PACs migrate towards the horizontal myoseptum and assemble into the longitudinally aligned PL^44^. This migration and aligning process requires guidance by muscle pioneers (MP)^45^, rostral primary motoneurons (PMN)^45^,^46^ and dorsal root ganglia (DRG)^47^. The impairment of *junb* or *miR-182* morphants in forming the PL may be due to (i) failure in LEC specification, (ii) defective sprouting angiogenesis from the PCV, (iii) impairment in the dorsal extension of the secondary ISV sprouts, or (iv) failure in the turning of the sprouts towards the horizontal myoseptum. Currently, we cannot rule out that Junb and its target miR-182 are implicated in LEC specification as *junb* expression has been observed in the gastrointestinal tract of zebrafish^27^ (see also Supplementary Fig. S1). Recently, Wnt5b has been reported as novel lymphatic inductive signal acting via β-catenin/TCF activation^43^. Interestingly, the Junb sibling c-Jun has been shown to act as a scaffold in the β-catenin–TCFs transcription complex bridging Dishevelled to TCF in zebrafish development and mammalian cells upon Wnt signals^49^. Due to the high conservation of c-Jun and Junb it is conceivable that Junb could play a similar role in Wnt5b signalling in PCV angioblasts. In tumour cells, β-catenin was previously shown to induce *miR-182,* which in turn leads to an increased invasion by targeting the matrix metalloproteinase inhibitor RECK^40^. For the future, time-lapse studies in a reporter zebrafish line such as *Tg*(*fli1:gal4*^*ubc3*^*; UAS:Kaede*^*rk8*^)^50^ with genetically ablated *junb* or *miR-182* shall be helpful to decipher the involvement of Junb-miR-182 in LEC specification. Consequently, this phenotype is distinct from previously described zebrafish morphants displaying PL failure due to complete absence of vISVs as shown for *vegfc, vegfr3 and ccbe1*^51^ morphants.

In line with previous findings showing that *miR-182* is predominantly a neurosensory microRNA^36^,^37^,^38^, whole-mount *in situ* staining for *miR-182* in 30 hpf embryos, just before the PACs start migrating^44^, revealed expression in the eye, the optic tectum, and in addition in the notochord (see Supplementary Fig. S5). Thus, it is feasible that the guidance of the lymphatic sprouts towards the horizontal myoseptum may be hampered upon *miR-182* loss. Several studies have shown that extensive tissue cross talk is essential for this process^44^. Marker analyses for muscle pioneers, rostral primary motoneurons and dorsal root ganglia at 30 hpf, hence shortly before PL formation initiates as early as 32 hpf^52^,^53^, did not reveal any major morphological aberrations (see Supplementary Fig. S6) suggesting that the structural scaffold guiding lymphangioblasts *per se* is still present upon *junb* or *miR-182* knockdown. Nonetheless, it is possible that signalling events driven by these cell types fail to properly orchestrate the formation of the PL and subsequent TD in *junb* or *miR-182* morphants. Based on published data and our own findings we can tell that *junb*, *miR-182* and *foxo1* share common or neighbouring sites of expression, such as eye (*foxo1*^54^; *junb*^27^ *and miR-182*^36^, see also Supplementary Fig. S1 and Fig. S5, respectively), lateral line (*junbb*^55^ and *miR-182*^36^) and gut (*junb*^27^ and *foxo1*^56^) or notochord (*miR-182*, see Supplementary Fig. S5) versus hypochord (*foxo1*^56^), respectively. Due to the low expression of *junba and junbb* (see Supplementary Fig. S1) and *miR-182*^57^ (see Supplementary Fig. S5) in the trunk region during development, we were not able to decipher the exact site of *miR-182* action. Yet, with regard to increasing observations, that microRNAs can be released from their producer cell, circulate, re-enter another recipient cell and thereby act as mediators of cell-cell communication^58^,^59^, knowing the site of *miR-182* expression may be of secondary importance.

Therefore, we focused rather on the identification of downstream target(s), preferably a transcript of venous endothelial origin that should be overexpressed upon silencing of either *junb* or *miR-182* in zebrafish and thereby cause the PL phenotype. However, failure in zebrafish lymphangiogenesis has only been described within the context of knockdown approaches until now. An *in silico* search for validated miR-182 targets revealed *foxo1* as a putative valuable candidate. Foxo1 is the predominant Foxo transcription factor in endothelial cells^34^. Increased Foxo1 levels were shown to affect migration, sprouting and tube formation of ECs and thus, reducing the angiogenic potential of ECs^34^.

In the present work, we show that single knockdown of either *foxo1a* or *foxo1b* provoked a developmental delay at least until 48 hpf that was eventually compensated at 72 hpf. Knockdown of either *foxo1a* or *foxo1b* in *miR-182* morphants resulted in a significant amelioration of PL development from 8% to 32% and 40% embryos, respectively, with completely intact PL strongly indicating that Foxo1 is a physiological downstream target of the Junb/miR-182 axis. The inability to rescue PL formation completely upon *foxo1* co-silencing in *miR-182* morphants can be explained by our observations that mani-pulation of either *foxo1* paralog in zebrafish already caused a vascular developmental delay. Consequently, miR-182 is required to ensure precise Foxo1 levels that are a prerequisite for proper lymphatic vascular development in zebrafish. Therefore, miR-182 can be considered as an additional lymphangiomiR besides miR-31^60^. Yet, while miR-31 acts as a strong negative regulator by repressing *Prox1* in blood endothelial cells^60^, we identified miR-182 as a fine-tuner regulating lymphangiogenesis by attenuating *foxo1* expression. Moreover, while several miRNAs have been identified to modulate lymphangiogenesis by targeting of *Prox1*^60^,^61^,^62^ or *Vegfr3*^63^, miR-31^60^ and miR-182 are the only lymphangiomiRs with a proven *in vivo* function in zebrafish reported so far.

Taken together, our data identify Junb as transcription factor regulating lymphangiogenesis in zebrafish and the Junb/miR-182/Foxo1 axis as first example for the negative action of Junb through a miRNA-mediated suppression of a downstream transcription factor. Interestingly, similar to the physiological consequences of *Junb* ablation in mice^3^,^4^, complete as well as endothelial cell-specific ablation of *Foxo1* in mice resulted in embryonic lethality around E10.5 due to vascular remodelling defects^64^,^65^. Due to their severe impact on the development of the blood vasculature, neither Junb nor Foxo1 has been associated with lymphangiogenesis so far. Thus, it will be highly interesting to investigate in future stu-dies whether this Junb/miR-182/Foxo1 regulatory axis is equally important in mammalian development.

## Materials and Methods

### Cell culture

Generation and culture of mouse embryonic fibroblasts^22^, vascular smooth muscle cells^6^ and lung microvascular endothelial cells^4^ was performed as described previously.

### Cloning of *Junba*:EGFP and *Junbb*:EGFP reporter constructs

Primers were designed to flank the part of the 5′UTR and the CDS of zebrafish *junba* and *junbb* containing both morpholino binding sites (MO1, MO2) targeted in this study. The resulting 0.1 kb segment was cloned into pGEM-T, and further subcloned in-frame into pEGFP-N1 to generate a fusion protein. For primer sequences see Supplementary Table 7.

### Zebrafish lines

Embryos of the *Tg*(*fli1:EGFP*)^*y1*^ line^66^ and AB wild type were raised and staged as described^67^. Embryos were kept in E3 medium (5 mM NaCl, 0.17 mM KCl, 0.33 mM CaCl_2_, 5–10% methy-lene blue) at 28.5°C with or without 0.003% 1-phenyl-2-thiourea (Sigma) to suppress pigmentation and staged according to somite number or hours post-fertilization (hpf). All experimental procedures on animals were approved by the local government authority, Regierungspräsidium Karlsruhe (license no.: 35-9185.64) and carried out in accordance with the approved guidelines.

### Microinjection

Morpholinos (Gene Tools) or a mixture of CRISPR gRNAs and Cas9 mRNA were diluted in 0.1 M KCl and injected through the chorion of one-cell or two-cell stage embryos. Amount of gRNAs used was for either Junba or Junbb 250 pg or for both a combination of 200 + 200 pg each. Equal amounts of control CRISPR gRNA (derived from original pT7-gRNA), 5-mismatch miRNA morpholino (*miR-182*-5MM-MO) or standard control morpholino (std-MO) were used as negative controls, respectively. Morpholino sequences and injected amounts are provided in Supplementary Table 5. Dose escalation studies were performed to determine submaximal morpholino concentrations. To prevent p53-mediated induction of apoptosis in response to silencing of both *junba* and *junbb,* or of *foxo1a* and *foxo1b*, a *p53*-specific morpholino^68^ was co-injected at 1.5-fold dosis of the respective experimental morpholino. Yet, we did not observe a difference to embryos without *p53*-MO. Mimics were diluted in RNase-free water. 1 nl of 1–5 μM (8–40 pg) of miScript miRNA mimic (Qiagen) was injected into one-cell or two-cell stage embryos. AllStars Negative Control siRNA (Qiagen) was used as negative control for miRNA mimics. Sequences are given in Supplementary Table 6.

### Statistical analysis

Data are expressed as mean ± SD or SEM. Statistical analyses were carried out using SigmaPlot 12.3 (Systat Software Inc). The following statistical analyses were applied throughout the manuscript and were also specifically indicated in the respective figure legends: Limma for microRNA microarray analysis (For complete procedure, please refer to Supplementary Methods), unpaired Student’s t-test for quantitative qRT-PCR analysis and for pairwise comparisons of percentage vISV of control versus experimental group; Mann-Whitney U-test for PL and TD quantifications. Mann-Whitney U-test was chosen to compare two rank sum tests of two experimental groups. Results were considered statistically significant for *P* < 0.05.

For further details on experimental procedures, including, CRISPR/Cas9 Zebrafish Mutant Generation, T7E1 Mismatch Assay, High-Resolution Melting Analysis, quantitative morphological analysis, RNA isolation and analyses, whole-mount *in situ* hybridization and whole-mount immunofluorescence staining please refer to Supplementary Methods.

## Additional Information

**How to cite this article**: Kiesow, K. *et al.* Junb controls lymphatic vascular development in zebrafish via miR-182. *Sci. Rep.* **5**, 15007; doi: 10.1038/srep15007 (2015).

## Supplementary Material

Supplementary Information

Supplementary Video 1

Supplementary Video 2

Supplementary Video 3

Supplementary Video 4

Supplementary Video 5

Supplementary Video 6

Supplementary Video 7

Supplementary Video 8
