# Supplementary material for: Junb controls lymphatic vascular development in zebrafish via miR-182
Source: Sci Rep. 2015 Oct 13;5:15007. doi: 10.1038/srep15007 (PMC4602192; doi:10.1038/srep15007)
Supplement: Supplementary Information [file srep15007-s1.pdf]

Supplementary Information:

Junb controls lymphatic vascular development in zebrafish via miR-182

Kristin Kiesow[1], Katrin Bennewitz[2,3], Laura Gutierrez Miranda[1], Sandra J. Stoll[2,3], Bettina Hartenstein[1], Peter Angel[1], Jens Kroll[2,3+*], Marina Schorpp-Kistner[1+*]

[1] Division of Signal Transduction and Growth Control, DKFZ-ZMBH Alliance, German Cancer Research Center (DKFZ), Heidelberg, D-69120, Germany, [2] Department of Vascular Biology and Tumor Angiogenesis, Center for Biomedicine and Medical Technology Mannheim (CBTM), Medical Faculty Mannheim, Heidelberg University, Mannheim, D-68167, Germany, [3] Division of Vascular Oncology and Metastasis, German Cancer Research Center (DKFZ), Heidelberg, D-69120, Germany

[+] these authors contributed equally to this work and are co-senior authors

Supplementary Data:

Supplementary Figure S1 to S6

Supplementary Tables 1 to 7

Supplementary Video Legends

Supplementary Materials and Methods

Supplementary Videos 1 to 8

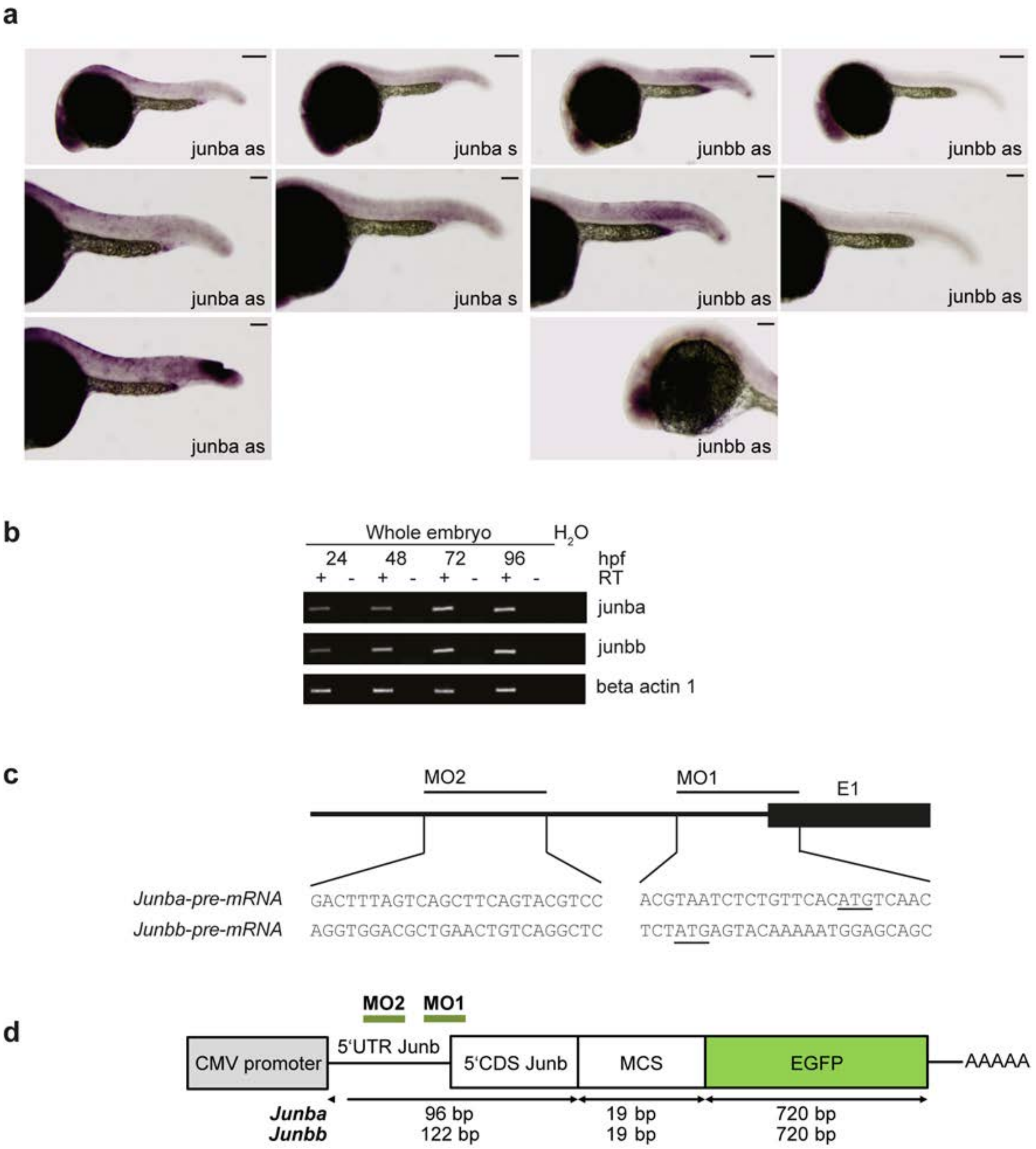

Supplementary Figure S1 (related to Figure 2). Expression of *junb* in zebrafish embryos and *junb* morpholino design and its validation. (a) Whole-mount *in situ* hybridization of 24 hpf embryos shows expression of *junba* and *junbb* mRNA in the eye an danal placode. *Junbb* mRNA can also be detected in the mesoderm and vasculature. Staining with sense control probes is negative. Bottom panels show positive controls for detection of *junb* at sites of injury (left panel) and in the eye (right panel). Size bars correspond 250 µm in top panels, and 125 µm in middle and bottom panels. (b) Total RNA was isolated from developing zebrafish embryos at 24, 48, 72, and 96 hpf and used to determine transcript levels of *junba* and *junbb*

by semi-quantitative PCR. *Actb1* served as loading control. (c) Upper panel: *junb*-MOs were designed to block translation of *junba* and *junbb* mRNAs due to the lack of exon-intron boundaries. Lower panel: gene sequences of regions targeted by *junba*-MO1/MO2 and *junbb*-MO1/MO2 around or upstream of start of translation. Start codon is underlined. (d) Schematic depiction of the 0.1 kb-long *junb*:EGFP fusion constructs created to determine the knockdown efficiency of the *junb*-MOs. A 0.1 kb segment of the 5'UTR/5'CDS of zebrafish *junba* or *junbb* carrying the binding sites for both experimental morpholinos (green line: MO1, MO2) was amplified from genomic zebrafish DNA, subcloned into pGEM-T, and further cloned in-frame into pEGFP-N1.

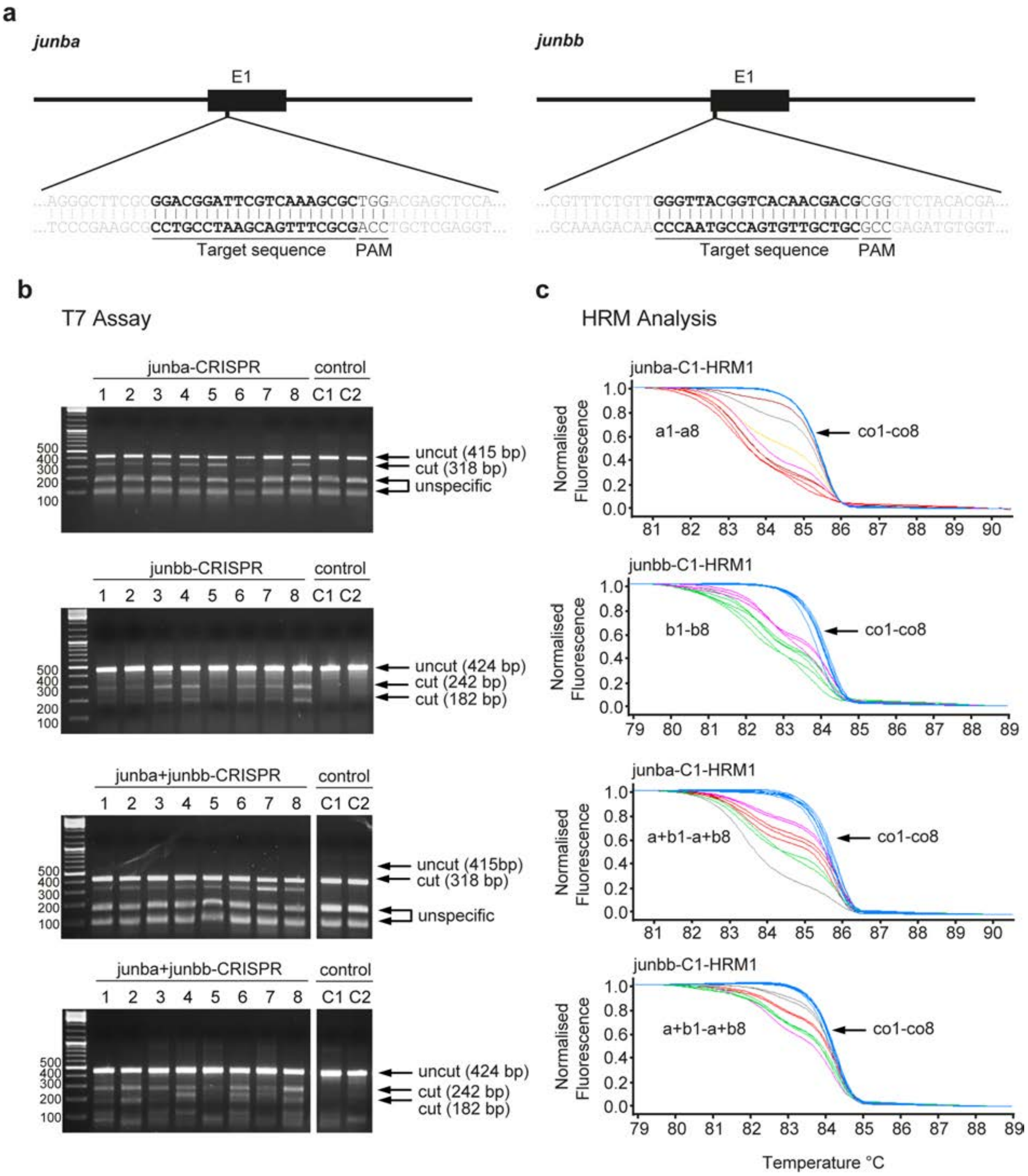

Supplementary Figure S2 (related to Figure 3). Validation of *junba* and *junbb* double knockout in zebrafish. (a) Schematic illustration of *junb* CRISPR target regions (bold) and protospacer adjacent motif (PAM, black) within the single-exon genes *junba* and *junbb*. (b) Identification of embryos with CRISPR/Cas9-mediated indel mutations by T7 endo I assay. Eight randomly selected embryos injected with CRISPR gRNA as indicated on the top were

analysed for presence of mutations at the CRISPR target regions of *junba* and *junbb*. DNA from single embryos injected with indicated *junb* - or control CRISPR was PCR amplified with *junba* - or *junbb* -C1-T7 primer, respectively, hybridized with amplicons from uninjected controls and digested with T7 endo I. Arrows indicate expected position of DNA bands for wild type amplicon (uncut) and those cleaved by T7 (cut). Unspecific bands of 264 and 156 bp arise from a single nucleotide polymorphism within the *junba* gene and subsequent T7-mediated cleavage of the resulting heteroduplex PCR amplicons. (c) Identification of embryos with CRISPR/Cas9-mediated *junba* and *junbb* indel mutations with high resolution melting (HRM) analysis of 0.1kb-PCR amplicons covering the CRISPR target site. Melting curves normalized to control CRISPR gRNA-injected embryos denote several different lesions in *junba* - (a1-a8) and *junbb* - (b1-b8) single as well as *junba+junbb* double CRISPR gRNA-injected embryos (a1-a8, b1-b8, a+b1-a+b8, non-blue coloured lines) compared to control CRISPR-injected embryos (co1-co8, blue lines).

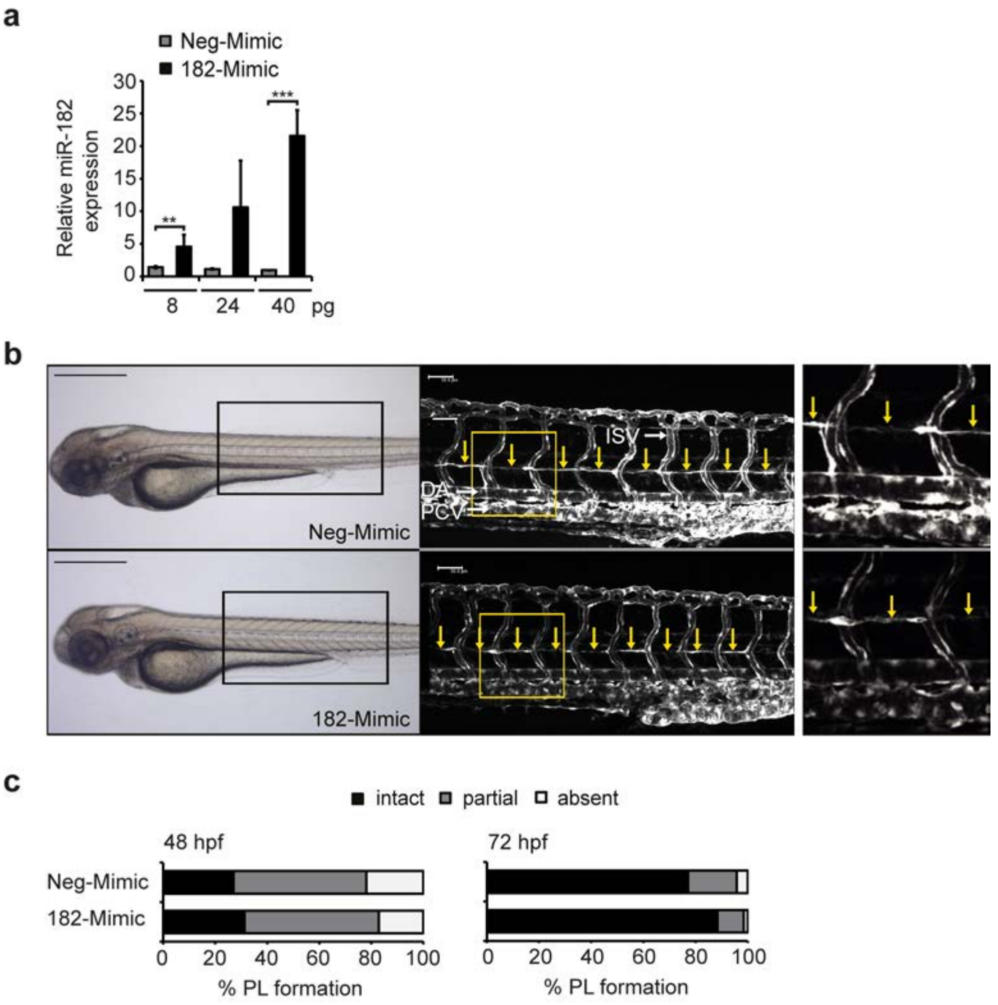

Supplementary Figure S3 (related to Figure 5). Ectopic administration of miR-182 does not cause vascular defects in zebrafish. (a) Overexpression of *miR-182* was assessed by Taqman miRNA assay in 48 hpf zebrafish embryos injected with miR-182-mimic (182-Mimic) or negative control mimic (Neg-Mimic) as indicated mean ± SD (n=3-5). *miR-182* expression level in 40 pg Neg-Mimic injected embryos was set to 1. *** $P < 0.001$, ** $P < 0.01$, Unpaired Student's t-test. (B) Left panel, bright field images of embryos injected with 40 pg Neg-Mimic (n=129) or miR-182-Mimic (n=114). Middle panel, confocal images of indicated trunk region in *Tg(fli1:EGFP)*^{y1} embryos at 72 hpf. Right panel, enlarged view of trunk region marked by the yellow rectangle in the middle panel. DA, dorsal aorta, PCV, posterior cardinal vein, and ISV, intersegmental vessels. PL is marked with yellow arrows or, if absent, with asterisks. Panels are lateral views, dorsal is up, anterior to the left. Black scale bar: 500 µm; white scale bar: 50 µm. (c) Percentage of PL defects as described in Figure 5.

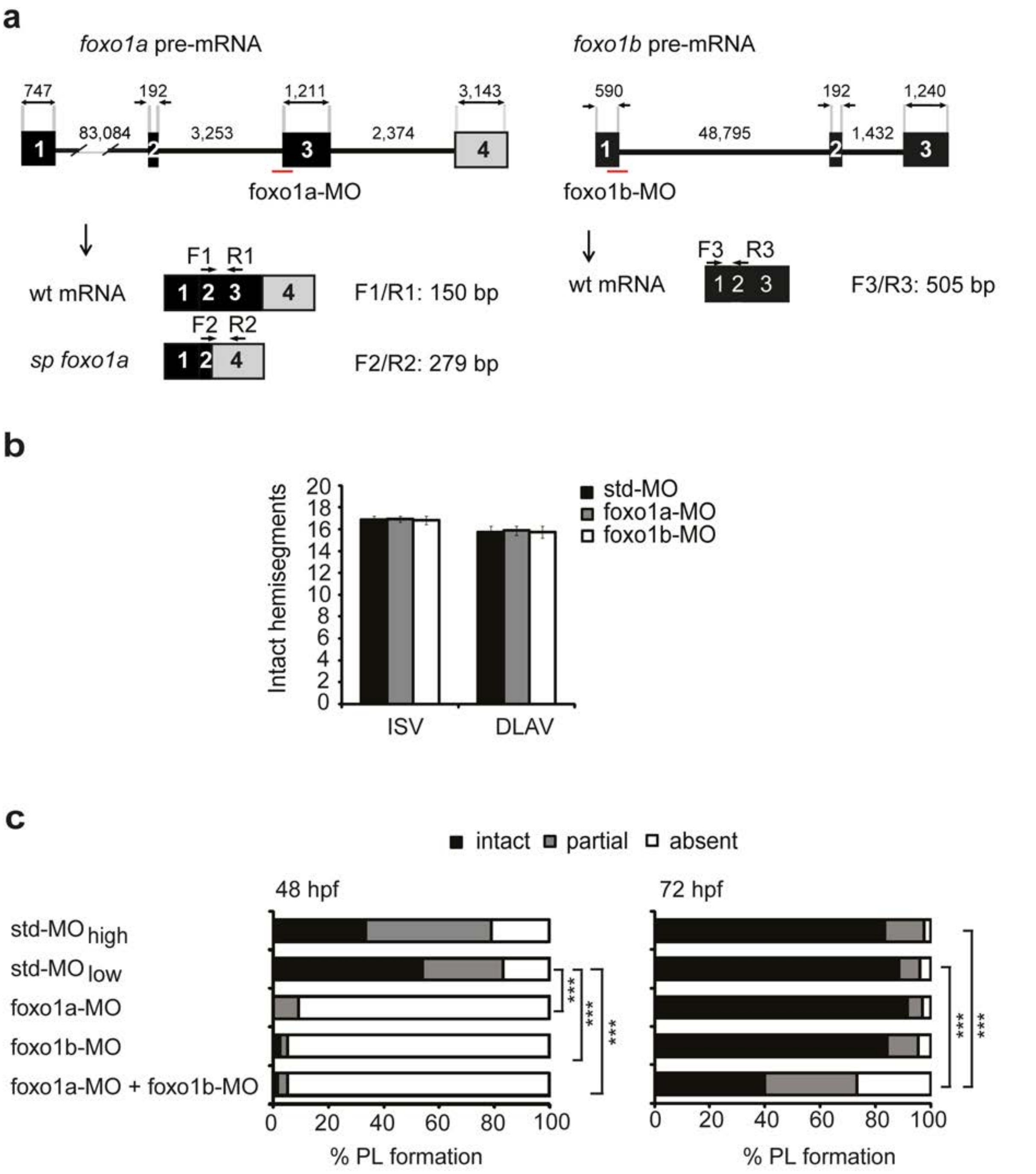

Supplementary Figure S4 (related to Figure 7). Knockdown of *foxo1* in zebrafish leads to mild developmental delay. (a) Partial pre-mRNA structure and splicing scheme (not to scale) for *foxo1a* (foxo1a-210, ENSDART00000171723) and *foxo1b* (ENSDART00000087896), showing introns (black bars), coding exons (black boxes), non-coding exon (grey box), size of exons in bp (numbers above), MO target sites (red bars) and primer pairs used for product amplification (black arrows). In contrast to the silencing

approach for the intronless *junba* and *junbb* genes, we selected here exon-intron blocking MOs to silence *foxo1a* and *foxo1b* transcripts. Silencing of *foxo1a* using a MO blocking at the intron2-exon3 splice site led to an exon-skipping splice product, referred to as *sp foxo1a* which can be detected by specific RT-PCR yielding an amplification product of 279 bp. Expression silencing of *foxo1b* using a splice-blocking morpholino at the exon1-intron1 splice site abolished any *foxo1b* products. RT-PCR using *foxo1b*-specific primers F3 and R3 yields a 505 bp product from std-MO targeted embryos, while no amplification product was obtained upon silencing with the *foxo1b*-MO. (b) Quantification of number of intact intersegmental vessels (ISVs) and dorsal longitudinal anastomotic vessel (DLAV) segments in zebrafish embryos injected with *foxo1a*-MO (2 ng, n=24), *foxo1b*-MO (2 ng, n=67), and std-MO (2 ng, n=40). (c) Quantification of PL defects of 48 hpf and 72 hpf embryos injected with std-MO (high: 14 ng, n=181; low: 4 ng, n=24), *foxo1a*-MO (2 ng, n=67), *foxo1b*-MO (2 ng, n=40), *foxo1a*-MO + *foxo1b*-MO (2+2 ng, n=178). *** $P < 0.001$, Mann-Whitney U-test.

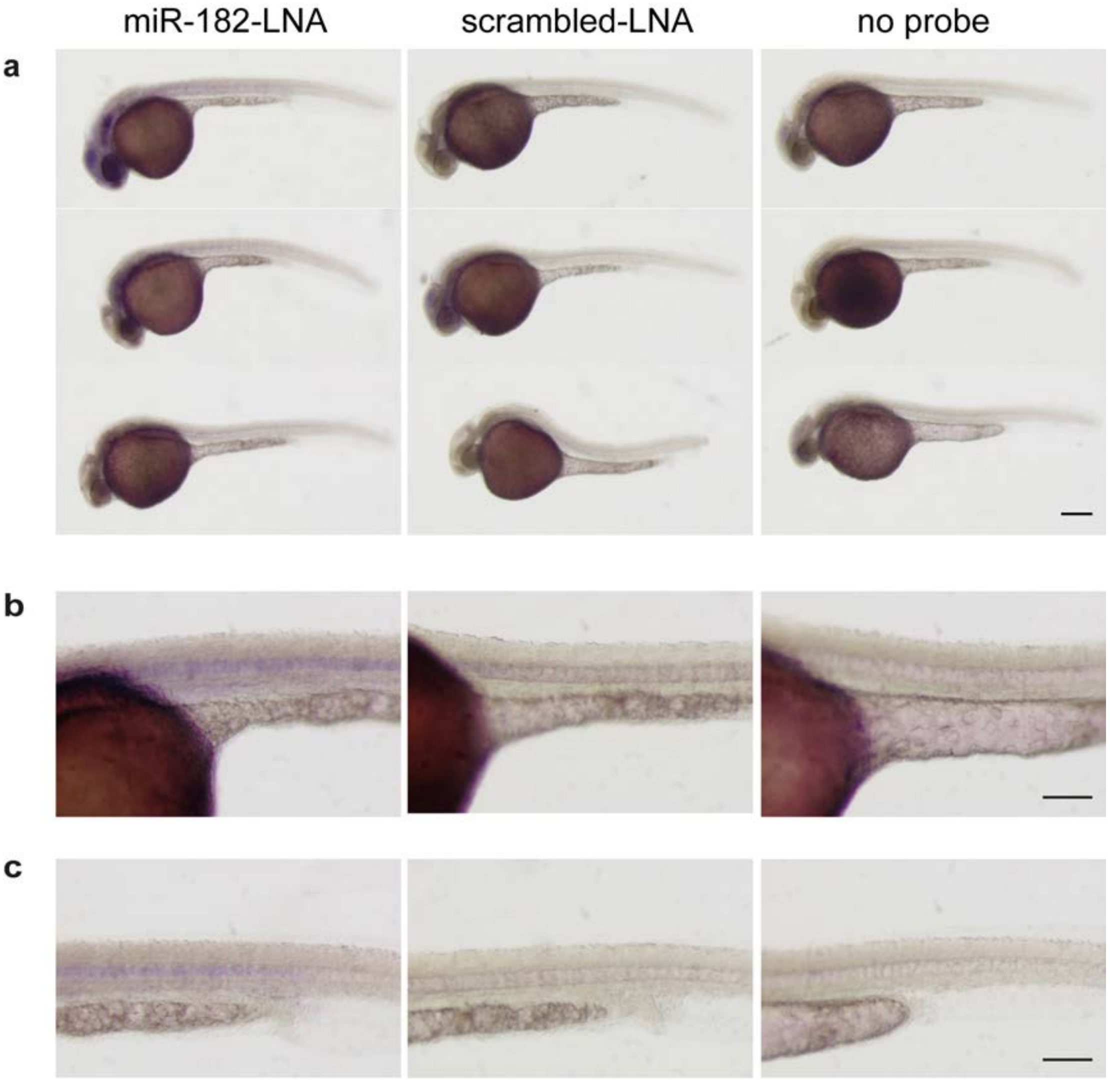

Supplementary Figure S5. Analysis of *miR-182* expression in 30 hpf embryos. (a-c) Whole-mount *in situ* hybridization of 30 hpf wild type embryos using miRCURY LNA™ microRNA Detection Probes for *miR-182* (miR-182-LNA, left panel) or a scrambled control (scrambled-LNA, middle panel). Right panel shows images of embryos incubated without LNA probe. (b) Higher magnification images of (a) showing the yolk sac and trunk region. (c) Higher magnification images showing soley the trunk region. *miR-182* expression can be detected in eye lens, olfactory placode, optic tectum and notochord. Size bar corresponds to 250 μm in (a) and 125 μm in (b, c).

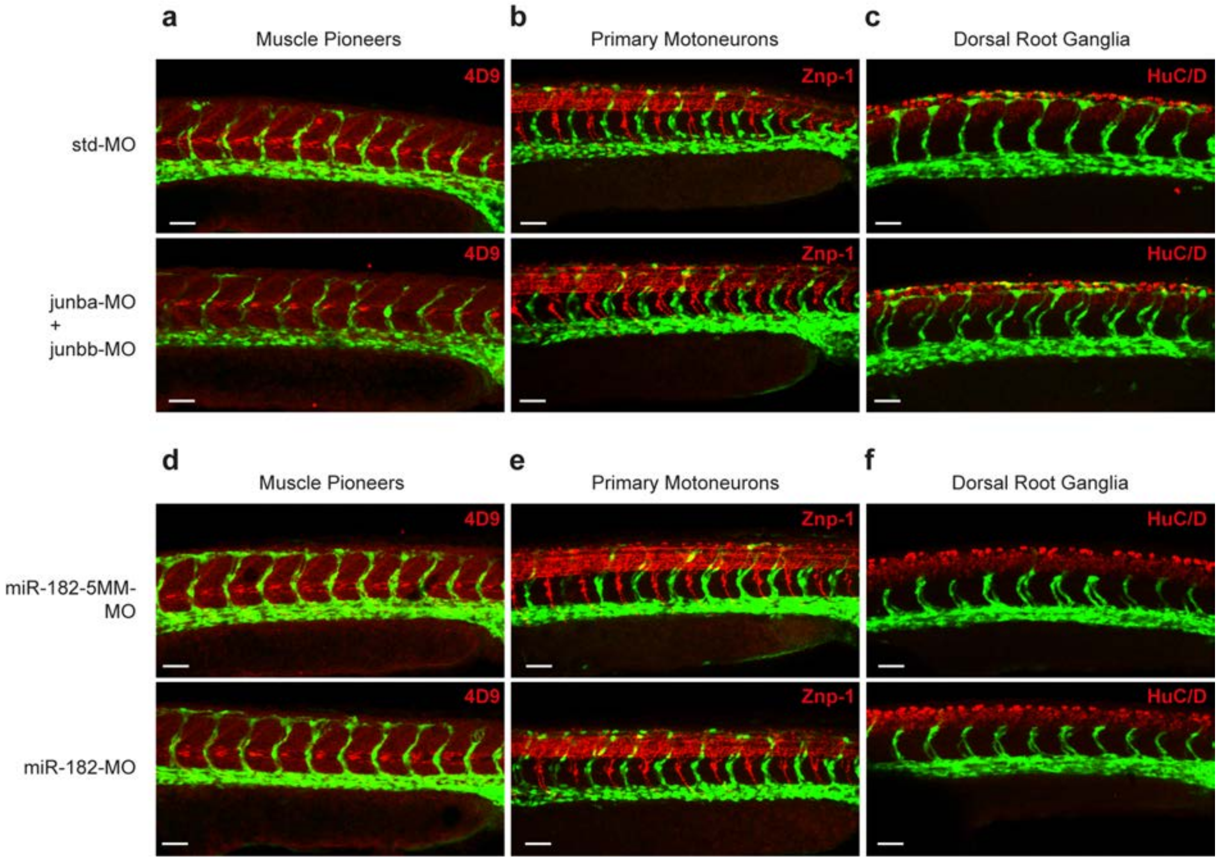

Supplementary Figure S6. Non-endothelial structures supporting LC formation are not altered upon Junb or miR-182 knockdown. (a-f) Whole-mount immunostaining of co-MO or *junba*- and *junbb*-MO1 (a-c), *miR-182*-5MM-MO or *miR-182*-MO-injected (d-f) *Tg(fli1:EGFP)*^{y1} embryos at 30 hpf for (a, d) muscle pioneers (4D9, red), (b, e) primary motoneurons (Znp-1, red) and (c, f) dorsal root ganglia (HuC/D, red). Images show confocal projections of the trunk vasculature of representative embryos. For each condition we examined a minimum of 5 embryos. Panels are lateral view, dorsal is up, anterior to the left. Overall morphology of muscle pioneers, primary motoneurons and dorsal root ganglia showed no alterations in *junba* + *junbb* or *miR-182* morphant embryos compared to their respective controls. White scale bar: 50 µM.

Supplementary Video Legends

Supplementary Video 1. In vivo imaging of the heart region of a representative zebrafish embryo at 48 hpf injected with a std-MO. Related to Fig. 2c.

Supplementary Video 2. In vivo imaging of the heart region of a representative zebrafish embryo at 48 hpf injected with *junba*-MO1. Related to Fig. 2c.

Supplementary Video 3. In vivo imaging of the heart region of a representative zebrafish embryo at 48 hpf injected with the *junbb*-MO1. Related to Fig. 2c.

Supplementary Video 4. In vivo imaging of the heart region of a representative zebrafish embryo at 48 hpf injected with the *junba*-MO1+ *junbb*-MO1. Related to Fig. 2c.

Supplementary Video 5. In vivo imaging of blood flow in the trunk region of a representative std-MO-injected zebrafish embryo at 72 hpf. Related to Fig. 2c.

Supplementary Video 6. In vivo imaging of blood flow in the trunk region of a representative *junba*-MO1 + *junbb*-MO1-injected zebrafish embryo at 72 hpf. Related to Fig. 2c.

Supplementary Video 7. In vivo imaging of blood flow in the trunk region of a representative *miR-182*-5MM-MO-injected zebrafish embryo at 72 hpf. Related to Fig. 4d.

Supplementary Video 8. In vivo imaging of blood flow in the trunk region of a representative *miR-182*-MO-injected zebrafish embryo at 72 hpf. Related to Fig. 4d.

Supplementary Table 1. *RT-PCR primer sequences*

Target	Gene Symbol	Primer Sequence	Amplicon Size
junb	*junba*	FOR 5'-TACACAGCGGCGACCGGAGA-3'	214 bp
		REV 5'-TCGGCGGGGGCATTTGGTTC-3'	
junba-like	*junbb*	FOR 5'-CGTGCTGACGACCCCCACAC-3'	206 bp
		REV 5'-CCGACTGCAGGGAGGAGCC-3'	
beta actin 1	*actb1*	FOR 5'-CTTGCGGTATCCACGAGAC-3'	429 bp
		REV 5'-GCGCCATACAGAGCAGAA-3'	
foxo1a ex1/ex2	*foxo1a*	FOR 5'-GAGCAGAGGACGAGCGGCAAAG-3'	150 bp
		REV 5'-GGTCGGAAAGCCGTCCAGGC-3'	
foxo1a ex1/ex3	*foxo1a*	FOR 5'-CGGGCGGCCTCCATGGACAA-3'	279 bp
		REV 5'-GCACAGGCACCTGGTTTGGCA-3'	
foxo1b ex1/ex2	*foxo1b*	FOR 5'-AGCAGCCGGGCAACTCGAAC-3'	505 bp
		REV 5'-TGGACGCGCACAAAGCGACT-3'	
Foxo1	*Foxo1*	FOR 5'-ATGCTCAATCCAGAGGGAGG-3'	183 bp
		REV 5'-ACTCGCAGGCCACTTAGAAAA-3'	
beta actin	*actb*	FOR 5'- CGAGCAGGAGATGGGAACC -3'	102 bp
		REV 5'-CAACGGAAACGCTCATTGC -3'	
kdrl	*kdrl*	FOR 5'-GATGGAGATACACACCTTCAG-3'	109 bp
		REV 5'-TGCGTACCGATGACACATTTC-3'	
znp-1	*syt2a*	FOR 5'-GAGGAGAGAAACAGGAAGAGGA-3'	173 bp
		REV 5'-AAGATCAGCAGCTTGCAGGA-3'	
Hprt1	*hprt1*	FOR 5'-CCGCTGGACCTGTTTACATT-3'	156 bp
		REV 5'-CTTTAGCACGCACAGGACAA-3'	
18s rRNA	*18s rRNA*	FOR 5'-TCGCTAGTTGGCATCGTTTATG-3'	62 bp
		REV 5'-CGGAGGTTCGAAGACGATCA-3'	

Supplementary Table 2. *CRISPR gRNA Oligos*

CRISPR	Gene Symbol	Primer Sequence
Junba-C1	*junba*	FOR 5'- TAGGACGGATTCGTCAAAGCGC-3'
		REV 5'- AAACGCGCTTTGACGAATCCGT -3'
Junbb-C1	*junbb*	FOR 5'- TAGGGTTACGGTCACAACGACG -3'
		REV 5'- AAACCGTCGTTGTGACCGTAAC -3'

Supplementary Table 3. *CRISPR T7 Primers*

CRISPR	Primer	Primer Sequence
Junba-C1	Junba-C1-T7	FOR 5'-GGTAACGGCGTCATCACATC-3'
		REV 5'-CTTTCAGAGTAACGAGCCGC-3'
Junbb-C1	Junbb-C1-T7	FOR 5'- AAAACGCCGCTTCATTGACT-3'
		REV 5'-TCATCCAGAGCCTTGACGAA-3'

Supplementary Table 4. *CRISPR HRM Primers*

CRISPR	Primer	Primer Sequence
Junba-C1	Junba-C1-HRM1	FOR 5'-CGGGGCAGTATTTGTACGGT-3'
		REV 5'-CGGGGGCATTTGGTTCATTT-3'
Junbb-C1	Junbb-C1-HRM1	FOR 5'-ATGGAGCAGCCGTTTTACCA-3'
		REV 5'-CACGTTCAAGTTCATGCCCG-3'

Supplementary Table 5. *Morpholino sequences*

Morpholino	Dosis	Sequence
miR-182-MO	12 ng	5'-AGTGTGAGTTCTACCATTGCCAAAT-3'
miR-182-5MM-MO	12 ng	5'-AGTGTCACTTCTAGCATAGGCAAAT-3'
junba-MO1	2 ng	5'-GTTGACATGTGAACAGAGATTACGT-3'
junba-MO2	2 ng	5'-GGACGTACTGAAGCTGACTAAAGTC-3'
junbb-MO1	2 ng	5'-GCTGCTCCATTTTTGTACTCATAGA-3'
junbb-MO2	2 ng	5'-GAGCCTGACAGTTCAGCGTCCACCT-3'
foxo1a-MO	2 ng	5'-TGAAGAGCCAGCTATTAAGAGAGTA-3'
foxo1b-MO	2 ng	5'-CTTTGAGGGCCATTACCTTCCAGCC-3'
p53-MO	1.5-fold	5'-GCGCCATTGCTTTGCAAGAATTG-3'
std-MO	accordingly	5'-CCTCTTACCTCAGTTACAATTTATA-3'

Antisense morpholinos for *junba* and *junbb* were directed against the translational start site, those for *foxo1a* and *foxo1b* against splice sites.

Supplementary Table 6. *Information on miRNA-Mimics*

Mimic	Type	Dosis
miR-182-Mimic	Syn-dre-miR-182 miScript miRNA Mimic<br>5'-UUUGGCAAUGGUAGAACUCACACU-3'	40 pg
Neg-Mimic	AllStars Negative Control siRNA (Qiagen)	40 pg

miRNA mimics were purchased from Qiagen (Hilden, Germany).

Supplementary Table S7. *pEGFP-Zf-Junb-MO-Reporter Cloning Primers*

Gene Symbol	Reporter Plasmid	Primer Sequence
junba	*junba*-EGFP	FOR 5'-GCCGGAATTCAACGGAGAGAACTTGCGGAC-3'
		REV 5’-GCCGGGATCCTCGTCATAAAACGGTTGCTCC-3’
junbb	*junbb*-EGFP	FOR 5'-GCCGGAATTCTCGTTATTCGCGCTCCTTCT -3'
		REV 5’-GCCGGGATCCTCGTGGTAAAACGGCTG-3’

Supplementary Methods

CRISPR/Cas9 Zebrafish Mutant Generation. CRISPR target sites were identified and selected using ZiFiT Targeter [1]. CRISPR guide RNAs were cloned into pT7-gRNA plasmid provided from Wenbiao Chen[2]. CRISPR target sites are denoted in Supplementary Figure S2. Oligonucleotide sequences for cloning of CRISPR gRNAs are listed in Supplementary Table 2. gRNA from an empty pT7-gRNA is referred to as control CRISPR. For Junba (250 pg, n=68), Junbb (250 pg, n=94) or in combination against both (200+200 pg, n=150) versus embryos injected with control CRISPR at corresponding doses of 250 pg (n=99) or 400 pg (n=105) Each gRNA was injected together with 400 pg Cas9 mRNA.

T7E1 Mismatch Assay. T7 endonuclease I assay was used to detect mutations at CRISPR-target loci. 72 hpf embryos were lysed in 20 µl lysis buffer (10 mM Tris, pH8.0, 1 mM EDTA, 0.3% Tween, 0.3% glycerol), heated for 10 min at 98°C and digested with 10 mg/ml proteinase K for minimum 4h at 55°C for isolation of genomic DNA. A genomic region surrounding the CRISPR-target site was PCR amplified using RedLoad Taq Master Mix (Jena Bioscience) and purified using Qiaquick PCR purification kit (Qiagen). Amplicons generated from experimental or control CRISPR gRNA-injected embryos were hybridized with amplicons from control CRISPR gRNA-injected embryos, respectively, and digested with T7 endonuclease (NEB) as described in [3]. Digestion products were resolved on a 2% agarose gel. A comprehensive list of primers used for amplification prior to T7 digestion is provided in Supplementary Table 3.

High-Resolution Melting Analysis. Mutations in CRISPR gRNA-injected embryos were identified by HRMA essentially as described in [2]. For this, PCR products spanning the CRISPR target site were amplified from gDNA in High Resolution Melting Master Mix (Roche) with HRM qPCR primers (sequences provided in Supplementary Table 4) and

resulting amplicons were examined by high-resolution melting analysis on a Light Cycler 480 II (Roche) with the Gene Scanning Software (Roche).

Microscopy. *Tg(fli1:EGFP)*^{y1} embryos were manually dechorionated and anaesthesized with 0.05 % tricaine (Sigma). Morphological analysis of arterial and venous intersegmental vessels was performed using a CTR 6000 microscope (Leica) for analysis. For *in vivo* imaging, dechorionated and anaethesized embryos were embedded in 1% low melting point agarose (Promega) dissolved in 0.003 % PTU-supplemented E3 medium. Confocal imaging was performed using a TCS SP5 system (Leica). A movie showing blood circulation was recorded with a digital camera (Leica) mounted on a CTR 6000 microscope (Leica).

Quantitative morphological analysis. The number of intact intersegmental vessels (ISV) and dorsal longitudinal anastomotic vessel connections (DLAV) between the investigated ISVs in the trunk vasculature was determined at 48 hpf. 17 consecutive ISVs and 16 DLAV connections per embryo were analyzed for the anterior part between head and anus starting at the 6th ISV. For the calculation of the arterious-venous (A:V) ratio, the identity of arterial-venous ISVs was determined for 58 - 60 embryos at 72 hpf for 14 ISVs, starting at the 6th ISV anterior. Arteries and veins were distinguished based on vessel origin (aISV: DA, vISV: PCV) and blood flow directionality (aISV: upwards, vISV, downwards). The appearance of the parachordal lymphangioblasts (PL) was quantified in 70 - 150 embryos at 48 hpf and 72 hpf by scoring 16 consecutive segments between the ISVs with regard to the partial or complete absence of parachordal lymphangioblasts. To quantify thoracic duct (TD) formation we examined 10 consecutive somite segments (from somite 5 to 15) of 60 larvae for each experimental group for presence, partial (10%-90%) of segments show TD formation) or complete absence of the TD at 5 dpf.

RNA isolation and analyses. Total RNA was isolated from cells and dechorionated zebrafish embryos using TRIZOL® reagent or miRNeasy Mini/Micro Kit (Qiagen) according to the

manufacturer's protocol. Total RNA extracted from MEFs was subjected to microRNA profiling using the "Geniom® Biochip MPEA mus musculus" (Febit) with sequences as annotated in Sanger miRBase 14.0 [4]. For microarray data analysis, the limma software package was applied that uses moderated *t* and *F*-statistics and in addition borrows information from other genes for estimation of variences and standard errors of a single gene [5]. Thereby, the analysis for particular small sample sizes is stabilized. Limma is commonly used for miRNA expression analyses. The resulting *P*-values were adjusted for multiple testing by the Benjamini-Hochberg method [6,7]. A log fold change for a deregulated miRNA with a limma adjusted $P<0.05$ was considered statistically significant. miRNA expression data can be accessed via http://www.ncbi.nlm.nih.gov/geo/query/acc.cgi?token=jrkpfgomsecwmba&acc=GSE43380.

Expression of pri-miRNAs and mature miRNAs was quantified using TaqMan® Pri-miRNA and MicroRNA Assays (Applied Biosystems), respectively. For RT-PCR or quantitative PCR, 1-5 µg RNA was reverse transcribed into cDNA. using oligo-dT primer for RNA from cells or random hexamers for RNA from zebrafish embryos, respectively, and RevertAid M-MuLV reverse transcriptase (Fermentas) according to the manufacturer's instructions. PCR detection of *foxo1a* transcripts was performed with specific primer pairs as listed in Supplementary Table 1 and the following program: 94°C for 3 min (94°C for 45 s, 55°C for 45 s, 72°C for 1 min)×28, 72°C for 10 min). SYBR green-based real-time PCR was performed on the StepOnePlus real-time detection system (StepOne™ software v2.2, Applied Biosystems). Every PCR reaction was carried out in triplicates with 10 - 50 ng of cDNA in a final volume of 20 µl using Power SYBR® Green PCR Master Mix (Applied Biosystem). Since AmpliTaq polymerase (present in Power SYBR® Green PCR Master Mix) is optimized to work at 60°C, a two-step cycling stage was generally sufficient. An additional elongation phase was only required for > 300 bp amplicons. Primer specificity was assessed by melt curve analysis and amplicon size verification by agarose gel electrophoresis. Primer efficiency was determined

by analyzing consecutive two-fold dilutions of reference cDNA ranging from 50 ng to 0.1 ng expressing the gene of interest. Primer sequences are given in Supplementary Table 1.

The relative expression was calculated applying the ΔΔCT-method[8]. Thereby, variations in the initial amount of cDNA used were corrected by calculating normalized relative expression values. For this, cycles of threshold (CT) values for a gene of interest as well as for a housekeeping gene (*Hprt* or *β-Actin*) were acquired for the same sample.

Whole-mount *in situ* hybridization. For generation of ISH antisense and sense probes *junba* and *junbb* cDNA[9], respectively, were cloned into a pBSK vector and *in vitro* transcribed using DIG RNA labeling mix (Roche). Whole-mount *in situ* hybridization of dechorionated 30 hpf zebrafish embryos was perfomed according the protocol of Thisse and Thisse[10]. Embryos were treated with Proteinase K (10 µg/ml) for 8 minutes. Each RNA probe (100 ng) was hybridized with fish embryos at 70°C over night. BCIP/NBT staining was performed at 4°C over night. Stained embryos were imaged with a Leica M10 and a Olympus camera XC50 images were processed using Adobe Photoshop CS5.1 and Illustrator CS5.1software.

Whole-mount immunofluorescence staining of zebrafish embryos. Whole-mount immunostaining of *Tg(fli1:EGFP)*^{y1} embryos was performed as described previously [11] with modifications as follows: 30 hpf embryos were permeabilized with 10 µg/µl proteinase K for 20 min and incubated overnight at 4°C with anti-4D9 (1:50, 39.4D5, Developmental Studies Hybridoma Bank, DSHB, University of Iowa, IA, USA), anti-Znp-1 (1:100, Zebrafish International Resource Center, ZIRC, University of Oregon, OR, USA), anti-HuC/D (1:500, anti-Elav1, 16A11, Molecular Probes, Eugene, OR, USA) to stain for muscle pioneers, primary motoneurons and dorsal root ganglia, respectively. Immunostained structures were subsequently detected with Alexa-546 coupled secondary anti-mouse antibody (1:250, A10036, LifeTechnologies, Carlsbad, CA; USA). Immunostained embryos were mounted in

1% low melting point agarose dissolved in PBS in glass culture cylinders (14mm x 5mm, Bioptechs Inc., Butler, PA, USA) and imaged with a LSM700 confocal laser scanning fluorescent microscope (Zeiss, Jena; Core Facility LMF, DKFZ, Heidelberg, Germany) using a 10x/0.3 dry objective. Images were processed using ZEN (Zeiss, Jena, Germany), ImageJ 1.46r, IrfanView 4.35 and Adobe Photoshop CS5.1 software.

1 Herwig, L. *et al.* Distinct cellular mechanisms of blood vessel fusion in the zebrafish embryo. *Current biology : CB* **21**, 1942-1948 (2011).

2 Jao, L. E., Wente, S. R. & Chen, W. Efficient multiplex biallelic zebrafish genome editing using a CRISPR nuclease system. *Proc Natl Acad Sci U S A* **110**, 13904-13909 (2013).

3 Isogai, S., Lawson, N. D., Torrealday, S., Horiguchi, M. & Weinstein, B. M. Angiogenic network formation in the developing vertebrate trunk. *Development* **130**, 5281-5290 (2003).

4 Jones, D. *et al.* Mirtron microRNA-1236 inhibits VEGFR-3 signaling during inflammatory lymphangiogenesis. *Arteriosclerosis, thrombosis, and vascular biology* **32**, 633-642 (2012).

5 Smyth, G. K. Linear models and empirical bayes methods for assessing differential expression in microarray experiments. *Statistical applications in genetics and molecular biology* **3**, Article3 (2004).

6 Benjamini, Y. & Hochberg, Y. Controlling the False Discovery Rate - a Practical and Powerful Approach to Multiple Testing. *J Roy Stat Soc B Met* **57**, 289-300 (1995).

7 Hochberg, Y. A Sharper Bonferroni Procedure for Multiple Tests of Significance. *Biometrika* **75**, 800-802 (1988).

8 Pfaffl, M. W., Horgan, G. W. & Dempfle, L. Relative expression software tool (REST) for group-wise comparison and statistical analysis of relative expression results in real-time PCR. *Nucleic acids research* **30**, e36 (2002).

9 Ishida, T., Nakajima, T., Kudo, A. & Kawakami, A. Phosphorylation of Junb family proteins by the Jun N-terminal kinase supports tissue regeneration in zebrafish. *Dev Biol* **340**, 468-479 (2010).

10 Thisse, C. & Thisse, B. High-resolution in situ hybridization to whole-mount zebrafish embryos. *Nature protocols* **3**, 59-69 (2008).

11 Stoll, S. J., Bartsch, S., Augustin, H. G. & Kroll, J. The transcription factor HOXC9 regulates endothelial cell quiescence and vascular morphogenesis in zebrafish via inhibition of interleukin 8. *Circulation research* **108**, 1367-1377 (2011).
